# Virulotyping of *Salmonella enterica* serovar Typhi isolates from Pakistan: Absence of complete SPI-10 in Vi negative isolates

**DOI:** 10.1371/journal.pntd.0006839

**Published:** 2018-11-30

**Authors:** Sadia Liaquat, Yasra Sarwar, Aamir Ali, Abdul Haque, Muhammad Farooq, Ilargi Martinez-Ballesteros, Lorena Laorden, Javier Garaizar, Joseba Bikandi

**Affiliations:** 1 Enteric Pathogen Laboratory, Health Biotechnology Division, National Institute for Biotechnology and Genetic Engineering (NIBGE), Faisalabad, Pakistan affiliated with Pakistan Institute of Engineering and Applied Sciences (PIEAS), Islamabad, Pakistan; 2 Departments of Bioinformatics and Biotechnology, Government College University Faisalabad, Faisalabad, Pakistan; 3 Akhuwat Faisalabad Institute For Research In Science And Technology, Faisalabad, Pakistan; 4 Department of Immunology, Microbiology and Parasitology, University of the Basque Country, (UPV/EHU), Vitoria-Gasteiz, Spain; International Vaccine Institute, REPUBLIC OF KOREA

## Abstract

The pathogenesis of *Salmonella enterica* serovar Typhi (*S*. Typhi), the cause of typhoid fever in humans, is mainly attributed to the acquisition of horizontally acquired DNA elements. *Salmonella* pathogenicity islands (SPIs) are indubitably the most important form of horizontally acquired DNA with respect to pathogenesis of this bacterium. The insertion or deletion of any of these transferrable SPIs may have impact on the virulence potential of *S*. Typhi. In this study, the virulence potential and genetic relatedness of 35 *S*. Typhi isolates, collected from 2004 to 2013 was determined by identification of SPI and non-SPI virulence factors through a combination of techniques including virulotyping, Whole Genome Sequencing (WGS), and Variable Number of Tandem Repeats (VNTR) profiling. In order to determine the virulence potential of local *S*. Typhi isolates, 56 virulence related genes were studied by PCR. These genes are located in the core as well as accessory genome (SPIs and plasmid). Major variations among studied virulence determinants were found in case of SPI-7 and SPI-10 associated genes. On the basis of presence of virulence related genes, the studied *S*. Typhi isolates from Pakistan were clustered into two virulotypes Vi-positive and Vi-negative. Interestingly, SPI-7 and SPI-10 were collectively absent or present in Vi-negative and Vi-positive strains, respectively. Two Vi-negative and 11 Vi-positive *S*. Typhi strains were also analyzed by whole genome sequencing (WGS) and their results supported the PCR results. Genetic diversity was tested by VNTR-based molecular typing. All 35 isolates were clustered into five groups. Overall, all Vi-negative isolates were placed in a single group (T5) whereas Vi-positive isolates were grouped into four types. Vi-negative and Vi-positive isolates were mutually exclusive. This is the first report on the comparative distribution of SPI and non-SPI related virulence genes in Vi-negative and Vi-positive *S*. Typhi isolates with an important finding that SPI-10 is absent in all Vi-negative isolates.

## Author summary

The distribution of virulence factors in *S*. Typhi can vary in isolates from different geographical regions and can have significant effect on the disease control. In this study, we have checked the distribution of 56 reported virulence associated factors in 35 local isolates of *S*. Typhi to identify any variations that can help in designing effective control strategies for typhoid. We have identified four naturally occurring variants which are simultaneously lacking SPI-7 and SPI-10, two adjacently located pathogenicity islands on *S*. Typhi chromosome. These isolates are not producing Vi capsular antigen hence the Vi based vaccines will not be effective against them. These findings highlight the need to develop typhoid vaccines specifically effective in Pakistan.

## Introduction

Pathogenicity islands are distinct genetic components located on the pathogenic bacterial chromosomes. The pathogenesis of *Salmonella enterica* serovar Typhi (*S*. Typhi) is mainly accredited to the possession of horizontally acquired large DNA elements that transcribe in a coordinated manner to produce an array of symptoms for the onset of disease. *S*. Typhi is a human adapted pathogen. It causes a severe systemic infection, the typhoid fever, which is a serious worldwide public health problem. According to the World Health Organization (WHO) the annual global burden of typhoid fever is about 11–20 million new cases per year and 1% of which are fatal. More than 90% of typhoid fever cases occurred in Asia [1,2]. It is highly prevalent in Asia and Africa due to shortage of hygienic water and poor sanitation. It is also a significant travel-associated disease [3]. Therefore, *S*. Typhi infection poses substantial burden on healthcare system throughout the world especially in Southeast Asia (including Pakistan) and other endemic countries. Typhoid fever is clinically manifested by prolonged fever, abdominal discomfort, headache, and general lethargy. Early diagnosis and treatment using an appropriate antibiotic are essential for optimal management of typhoid fever, especially in children. Unfortunately, the emergence of multidrug-resistant *S*. Typhi strains causes difficulty in its treatment and poses a serious threat to future treatment options [4,5,6,7,8].

The complex pathogenesis of systemic *Salmonella* infections is associated with the presence of various defensive as well as offensive virulence factors. These factors contribute for its success as an intracellular human pathogen and participate at various stages of invasion, intracellular replication and survival within the host. Many of the *Salmonella* virulence genes are distributed on large genomic regions of 10–134 kb known as *Salmonella* pathogenicity islands (SPIs) [9]. The SPIs are characterized by a base composition different from the core genome and are often associated with *tRNA* genes and mobile genetic elements, like IS elements, transposons or phage genes [10]. Virulence factors encoded by SPI genes tamper with host cellular mechanisms and are thought to dictate the host specificity of different *S*. *enterica* serovars. Some virulence genes not located on SPIs such as the chromosomally-encoded *phoP/Q* (two component global regulator), *rpoS* (global stationary phase regulator) and *fliA* (RNA polymerase sigma factor for flagellar operon) also play important roles in the virulence of *Salmonella* [11,12].

Twenty one SPIs are known to date in *Salmonella* [13]. Out of these, 17 SPIs, 1 to 13 and 15 to 18 have been reported in *S*. Typhi. The largest of these islands, SPI-7 contains 134 kb of *S*. Typhi-specific DNA and carries biosynthesis genes (*viaB* locus) for the production of the Vi capsular antigen [14]. The Vi capsular antigen is a significant virulence factor for typhoid fever, as isolates positive for Vi production have higher rates of infection [15,16], and it continues to be the focus for prophylaxis for this disease. Volunteer studies have indicated that Vi-positive strains of serovar Typhi are more virulent in humans than Vi-negative isolates, although Vi production is not essential for the infection process in humans [17].

Vi-negative isolates (lacking Vi capsular polysaccharide antigen) of serovar Typhi have been reported in regions where typhoid fever is endemic. Previously, we have reported the existence of two types of naturally occurring Vi negative *S*. Typhi in Faisalabad region of Pakistan with partial (*viaB* operon only) or total absence of SPI-7 [18].

*S*. Typhi isolates which are genetically diverse with clonal expansion and genome variations have been reported in Malaysia and Southeast Asia. These genetic variations may be important for virulence [19,20,21,22]. The severity of the illness varies in different areas and this may be due to genetic diversity among the endemic strains [23]. Recent genome sequence projects demonstrated that *S*. Typhi strains showed limited genetic variation [24].

Genome sequence data on *S*. Typhi strains from different countries are required to clearly recognize their virulence potential. A well-known quick typing method is virulotyping that is used for detection and profiling in pathogenic bacteria. It increases our understanding of possible risk for human and animal infections. Virulotyping is a valuable tool for the characterization of *Salmonella* isolates [25]. In most of the studies involved in virulotyping, virulence factors with reported contributions to virulence were screened by PCR using gene specific primers. To find the presence of virulence genes, monoplex and multiplex PCR is routinely carried out. The distribution of SPIs has already been investigated in a reference strain CT18 [26] but such studies on naturally occurring Vi-positive and Vi-negative strains of *S*. Typhi are infrequent. This study was designed to find differences, if any, in the distribution of SPIs and related virulence factors as wells as non-SPI virulence factors of Vi-positive and Vi-negative isolates from Punjab, Pakistan. For this purpose, the comparative distribution of a significant number of virulence factors in clinical isolates of Vi-negative and Vi-positive *S*. Typhi collected from local sources was investigated.

## Results

### Distribution of virulence genes in Vi-positive and Vi-negative *S*. Typhi isolates

In this study 56 virulence related genes involved in mobility, secretion systems, metabolic regulation and toxin production were screened by PCR. The distribution of the virulence related genes among local isolates is presented in Table 1. In this study, *S*. Typhi isolates showed clearly distinct virulence-gene profiles: Vi antigen-positive and Vi antigen-negative according to the association of the virulence genes with SPI-7 and SPI-10.

**Table 1:** Distribution of virulence related genes of chromosomal (SPIs, Non-SPI) and extrachromosomal origin (plasmids) among local isolates of *S*. Typhi (in percent).

Location	Virulence Related Genes	Percentage of *S*. Typhi Strains Positive by PCR	
		Vi-positive n = 31	Vi-negative n = 4
SPI-1	*invA*, *prgI*, *hilA*, *sipA*, *prgH*	100	100
SPI-2	*spiC*, *sseB*	100	100
SPI-3	*mgtb*, *mgtC*, *nepI/gaiA*	100	100
SPI-4	*spi4d*, *orfL*	100	100
SPI-5	*pipB*, *pipD*, *sopB/sigD*	100	100
SPI-6	*tcf*, *safC*	100	100
SPI-7	*pilS*, *tviA*, *tviB*, *tviD-E*	100	0
SPI-7	*sopE*	100	100
SPI-8	STY3280, STY3282	100	100
SPI-9	*prtB*, STY-2875	100	100
SPI-10	*sefC*, *sefB*, *sefR*, *prpZ*, *prkY*, *prkX*	100	0
SPI-11	*pagC*, *pagD*, *msgA*, *cdtB*	100	100
SPI-12	*sspH2*	100	100
SPI-16	*gtrA*, *gtrB*	100	100
SPI-17	STY-2629	100	100
SPI-18	*clyA/sheA/hlyE*, *taiA*	100	100
Non-SPI	*agfA*, *stgA*, *fimA*, *staA*, *sifA*, *phoP*, *rpoS*, *fliA*, *tolC*, STY1460	100	100
*R27, *pHCM1	*trhW*	29	0
*pRST98	*spvB*, *spvR*	0	0

*Plasmids

#### Virulence determinant associated to core genome

Ten virulence related genes associated with core genome were included in the present work. These genes encode outer membrane protein (*tolC*), adhesion factors (*agfA*, *staA*, *stgA*, *sifA* and *fimA*), sigma factor (*rpoS*, *fliA*), virulence transcriptional regulatory gene (*phoP*) and peptidase (STY1450). Each of the studied *S*. Typhi isolate harbored all these core genome associated virulence genes. No variation was observed in the genes located on core genome of *S*. Typhi.

#### Virulence determinant associated to accessory genome

Virulence genes associated to fifteen SPIs (SPI-1-12, and SPI-16-18) previously reported to be related to virulence were investigated in this study. All of the major virulence markers located on SPI-1 to 6, 8, 9, 11, 12 and 16–18 (Table 1) were identified in 100% of local isolates. On the contrary, some of the virulence determinants located on SPI-7 (*pilS*, *tviA* and *tviB*) and all virulence determinants of SPI-10 (*sefBCR*, *prpZ*, *prkY* and *prkX*) were absent in 11% (n = 4) of *S*. Typhi isolates. Both *tviA* and *tviB* are required for Vi capsule synthesis. As these four isolates (11% of total isolates) were expected to be incapable of Vi expression, they were designated as ‘Vi-negative’. The SPI-7 associated phage-related *sopE* gene was detected in all *S*. Typhi isolates. However, partial deletion in *sopE* prophage was found in case of Vi-negative isolates.

#### Virulence related genes located on plasmids

Three virulence genes (*spvB*, *spvR* and *thrW*) located on plasmids were also studied and 71% of strains did not possess any plasmid-associated virulence gene. Only one plasmid-associated gene *trhW* (located on pHCM1; encodes plasmid transfer protein involved in fimbrial regulation) was detected in 29% of isolates. This virulence gene was only detected in Vi-positive isolates.

### Evaluating the presence or absence of SPI-7 and SPI-10 in Vi-negative isolates

#### Polymerase chain reaction

**Fig 1:**
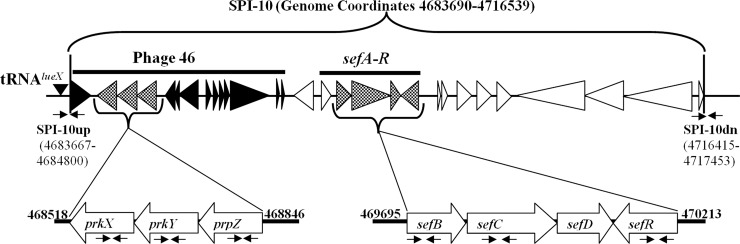
Physical map of the SPI-10 in the *Salmonella enterica* serovar Typhi CT18 NC_003198.1. Genes are depicted by arrows with gene designation indicated within arrows. Virulence genes are dotted and in black are phage-related genes. The schematic locations of primer pairs specific for SPI-10up, SPI-10dn, *prkX*, *prkY*, *prpZ*, *sefB*, *sefC*, and *sefR* are shown below genes. The position and length of expected PCR product (including region and genes investigated in this study) are mentioned according to the CT18 genome coordinates [26].

Presence or absence of complete SPI-7 and SPI-10 was searched using primers specific to their flanking sides. SPI-7 is flanked by two partially duplicated *tRNA*^*pheU*^ loci. Primers DE0032-F and DE0083-R have been previously used to demonstrate the lack of an insertion at the *tRNA*^*pheU*^ locus [27,28]. These primers generate a PCR amplicon of 1275bp if the island is absent. SPI-7 is 134kb in length; therefore, the presence of the island is outside the constraints of the PCR. All Vi-negative and Vi-positive isolates failed to give any amplification with these primers, suggesting the presence of SPI-7. It was found that in our all Vi-negative isolates SPI-7 was present but it lacked *viaB* and *pil* operons. The primer pair SPI10up-F, SPI10up-R, was designed to amplify the upstream flanking side whereas SPI10dn-F, SPI10dn-R for downstream flanking side of SPI-10. Fig 1 shows the position of these primers in the genome of *S*. Typhi CT18.

Interestingly, no PCR amplification was obtained with SPI-10 flanking side primers in case of Vi-negative isolates whereas Vi-positive isolates showed the respective amplifications for SPI-10 flanking side primers. The forward primer of upstream insertion site, SPI10up-F and reverse primer of downstream insertion site, SPI10dn-R were also used to amplify any genome sequences flanking the two insertion sites to verify the absence of SPI-10 but no amplification was observed. Therefore, it was concluded that SPI-10 was completely absent including the insertion sites from all our Vi-negative *S*. Typhi isolates. As expected, SPI-10 associated virulence genes (*sefBCR*, *prpZ*, *prkY* and *prkX*) were detected only in Vi-positive *S*. Typhi. In order to confirm the specificity of amplicons of SPI-10, associated virulence genes as well as amplified fragments of flanking sides of SPI-10 were sequenced. The sequences were then verified by BLASTn [29] against *Salmonella enterica* serovar Typhi sequences. All these sequences were confirmed as related to corresponding genes and region of SPI-10 of the *S*. Typhi genome.

#### Whole genome sequencing results

**Fig 2:**
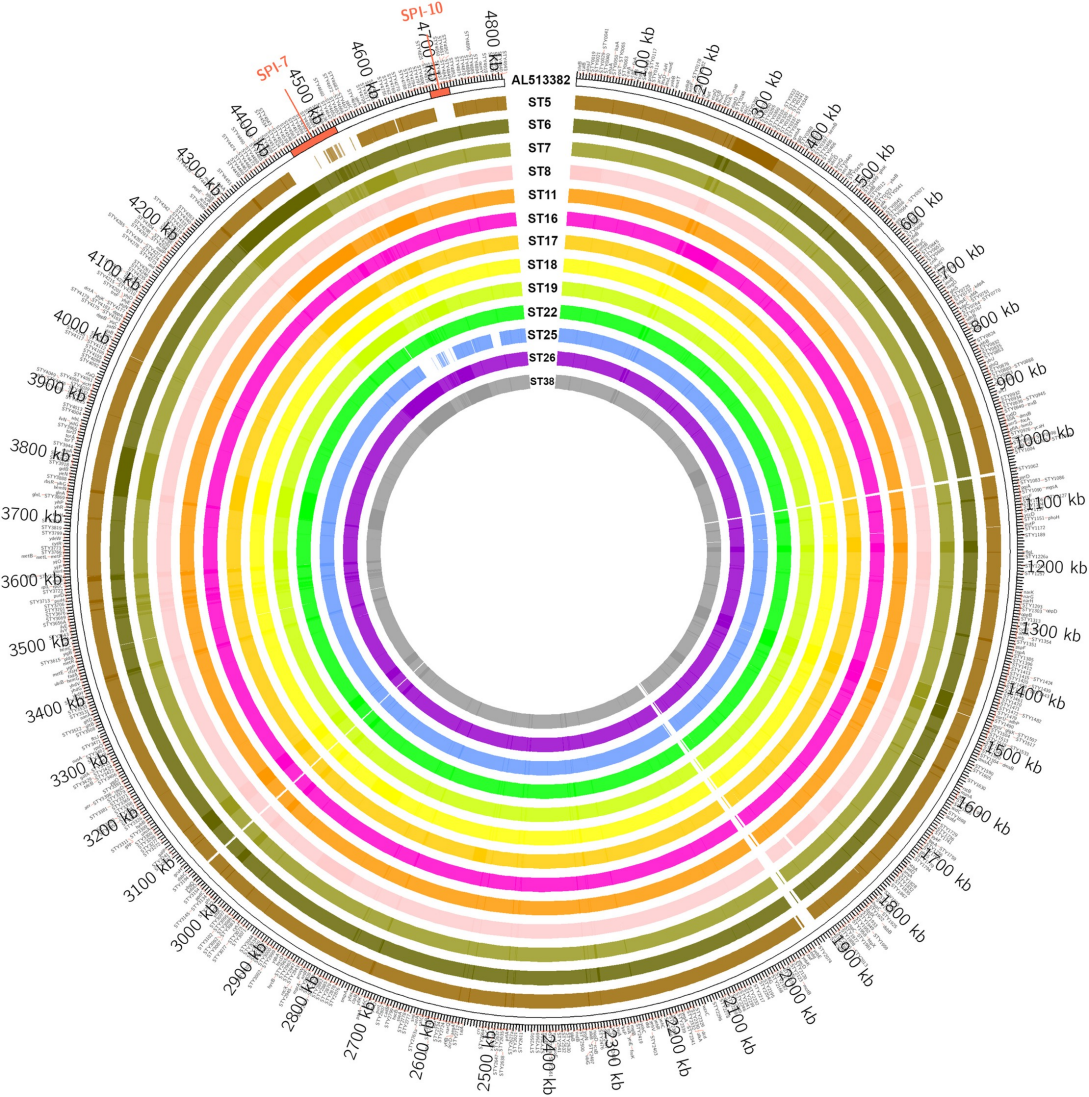
Comparative genome analysis of *Salmonella*Typhi strains for pathogenicity Islands. The outer circle corresponds to the reference genome of *S*. Typhi (Acc: AL513382) whereas inner circles depict the strains with clear depiction of absence of SPI-7 and SPI-10 in *S*. Typhi strain 5 and 25. The gene labels are displayed at 1500bp distance window size for figure adjustment.

Complete absence of SPI-10 in Vi-negative *S*. Typhi isolates was confirmed by the whole genome sequence analysis [29]. Altogether, 76.79 million read pairs of 100bp were obtained for the genome of 13 *S*. Typhi strains (2 Vi-negative and 11 Vi-positive) with an average of 5.9 million read pairs per sample, comprising average throughput of 1.18Gb per sample, as shown in supplementary table (S1 Table). All of the sequenced strains were having a minimum of 230x genome coverage, which is fairly good enough for a reliable comparison of bacterial genomes. The Vi-negative strains have 4062 complete and 49 partial genes (pseudogenes) as compared to the reference genes set (4395) of *S*. Typhi CT18 which is fairly low than the other Vi-positive strains. The NG50 statistics for the *de novo* assemblies is in the range of 145kb to 173kb. The results show that both Vi-negative strains (ST5 and ST25) have a higher level of divergence from the *S*. Typhi reference genome as the number of contig mismatches (>9300) and indels (>200) are higher than other strains, as shown in supplementary table (S1 Table). The comparative analysis of Nx, NGx, cumulative length, GC content and coverage histograms has been provided in supplementary figures S1 Fig–S31 Fig. Blastn [29] similarity search of assembled contigs with the *S*. Typhi reference genome confirmed the PCR findings that both Vi-negative strains (ST5 and ST25) did not have SPI-7 and SPI-10 except for some of the genes of SPI-7, as shown in Fig 2.

These data validate our hypothesis of simultaneous absence of SPI-7 and SPI-10 from Vi-negative isolates. The WGS data of the 13 isolates of *S*. Typhi have been submitted in GenBank and the accession numbers were assigned as SAMN08195664, SAMN08195665, SAMN08195666, SAMN08195667, SAMN08195668, SAMN08195669, SAMN08195670, SAMN08195671, SAMN08195672, SAMN08195673, SAMN08195674, SAMN08195675, SAMN08195676.

### Variable Number of Tandem Repeats (VNTR) based molecular typing of *S*. Typhi Local Isolates

**Fig 3:**
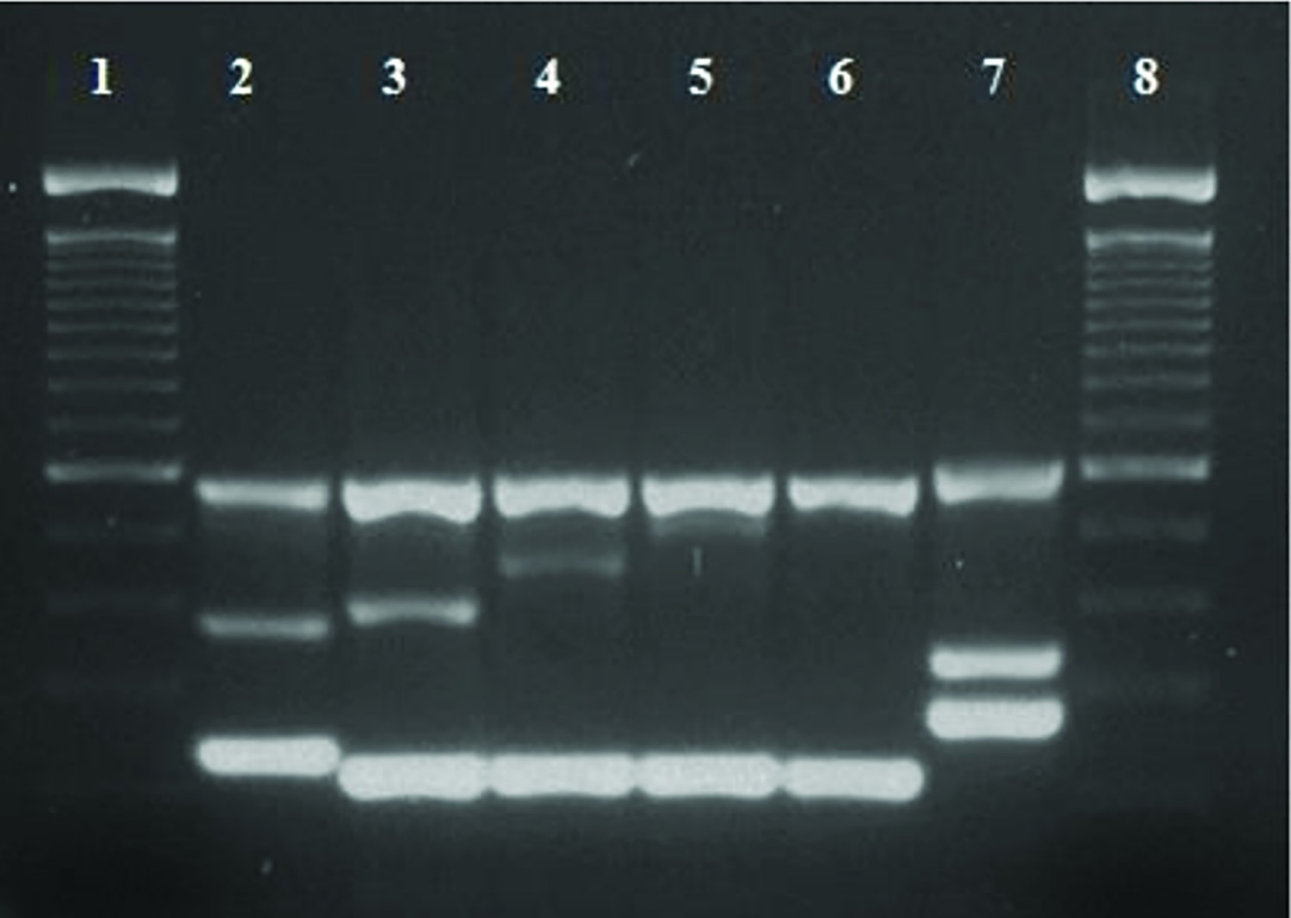
Agarose gel analysis of the all five types of VNTR profiles from representative *S*. Typhi local isolates. Lane 1 and 8:100bp DNA ladder (Invitrogen 15628–019; showing bands of 2072, 1500, 1400, 1300, 1200, 1100, 1000, 900, 800, 700, 600, 500, 400, 300, 200 and 100 bp), Lane 2–6: Vi-positive *S*. Typhi isolates, Lane 7: Vi- negative *S*. Typhi isolates.

On the basis of multiplex PCR results, all 35 *S*. Typhi isolates were grouped into 5 VNTR types (Fig 3). Overall, 3, 4 and 2 alleles were observed for TR1, TR2 and TR3, respectively. Therefore, all these nine type of amplicons from representative isolates were sequenced and number of repeats in each case was calculated by using tandem repeat finder (70). Table 2 briefly describes each observed VNTR type.

**Table 2:** VNTR profiles of 35 local isolates of *S*. Typhi.

Sr. #	VNTR profile (TR1/TR2/TR3)	No. of isolates (Vi+, Vi-)	VNTR profile designation
1	3.9x/10.5x/2.3x	15(Vi+)	T1
2	5x/13.9x/2.3x	6(Vi+)	T2
3	3.9x/12.5x/2.3x	6(Vi+)	T3
4	3.9x/ND/2.3x	4(Vi+)	T4
5	9.6x/3.1x/3.3x	4 (Vi-)	T5

ND (not detected); x = copy number

## Discussion

This study was focused on assessing the distribution of *Salmonella* pathogenicity islands (SPIs) in Vi-positive and Vi-negative variants of *Salmonella enterica* serovar Typhi and comparative virulotyping from local clinical samples. The virulence factors of the *Salmonella* serovars are mostly encoded by the *Salmonella* pathogenicity islands (SPIs). Two hallmarks of *Salmonella* pathogenesis, the host invasion and intracellular proliferation, are directly linked to the genes on SPIs. SPI-1 contains invasion genes whereas SPI-2 is required for intracellular pathogenesis. These SPIs encode two Type-III secretion systems (T3SS) and have crucial role for systemic *S*. *enterica* infections [30]. The *invA*, *hilA*, *sipA*, *prgl* and *prgH* genes are located on SPI-1 whereas *spiC*, *sifA* and *sseB* genes belong to SPI-2. In this study, we detected these genes in all *S*. Typhi isolates. SPI-3 is involved in intracellular survival and encodes a magnesium transporter (*mgtB* and *mgtC*) [31]. These genes were also detected in both types of isolates. Similar genetic homogeneity among Vi positive and Vi negative *S*. Typhi isolates was observed in virulence genes located on SPI-4 to 6, SPI-8, 9, 11, 12, and SPI-16-18. These findings are in accordance with previous studies [26,32].

Virulence factors on SPI-7 and SPI-10 are less stable than those associated with other SPIs [9]. In *S*. Typhi, SPI-7 is also referred as major SPI and represents the largest SPI with a size of 134 kb. It comprises of four parts: type IVB pilus operon, *sopE* prophage, Vi biosynthetic operon, and a 15 kb phage-like segment [26,27]. It codifies for the surface Vi polysaccharide antigen that contributes to virulence. However, lack of Vi expression can also be beneficial to some key steps of *S*. Typhi infectivity, for example, invasion, as Vi is the target of protective immune responses [33]. Vi-positive *S*. Typhi can benefit by inhibiting complement deposition at the bacterial surface and the post-phagocytic oxidative burst, thus resulting in reduced bacterial internalization and killing by phagocytes [34]. Serovar Typhi lacking Vi capsular polysaccharide antigen has been known and reported worldwide for several decades. Molecular evidence of the loss of Vi antigen has suggested that Vi-negative strains can be derived by the excision of SPI-7 or by a spontaneous base change in the *viaB* operon [28,35,36].

In the present study, all local isolate were carrying SPI-7 but some of them (11%) were deficient in SPI-7 associated genes (*tviA*, *tviB* and *pilS*). Results of this study indicated that 89% of analyzed *S*. Typhi isolates possessed *viaB* operon which is involved in biosynthesis of Vi-antigen. This virulence factor is also observed in *S*. Typhi isolates from typhoid patient’s blood, bone marrow as well as stool. It has been reported that most isolates were Vi-positive and only a few *S*. Typhi were Vi-negative [18,37,38]. The Vi-negative *S*. Typhi isolates that only lose expression of *viaB* operon have also been reported from India [37], and Nepal [38], but the absence of SPI-7 has been first time demonstrated in this study supported by whole genome sequencing (WGS).

SPI-10 is a pathogenicity island found next to the *tRNA*^*leuX*^ gene at centisome 93. In the *S*. Typhi genome, this island corresponds to a 33 kb fragment carrying a full P4-related prophage, termed ST46 and the *sefA-R* chaperone-usher fimbrial operon [26,39]. The *tRNA*^*leuX*^ region is a hypervariable hot spot for horizontal gene transfer in the *Salmonella* genus and different isolates from the same *S*. *enterica* serovar can exhibit significant variation in this region. Presence of mobile genetic elements and P4 phage play a major role in driving the variability of this region [39]. Genome sequence analysis identified three open reading frames carried by P4 like phage which have integrated within SPI-10 and are termed as *prpZ* gene cluster [39,40]. These are present in Ty2 and multi-drug resistant CT18 genomes of *S*. Typhi but absent in all other sequenced serovars of *S*. *enterica*. Several lines of evidence indicate that *S*. Typhi has acquired these three ORFs through Horizontal Gene Transfer (HGT). The *prpZ* gene cluster consists of three ORFs coding for proteins with homology to eukaryotic-type Ser/Thr protein phosphatases 2C (prpZ) and Ser/Thr protein kinases (prkY and prkX). It has been found to promote survival in macrophages [41,42].

The results in this study related to *prpZ* gene cluster were apparently distinct from previous reports [42,43,44]. Based on the findings of this study, we report for the first time that all our naturally occurring Vi-negative *S*. Typhi are lacking SPI-10. None of SPI-10 associated virulence genes (*sefB*, *sefC*, *sefR*, *prpZ*, *prkX*, and *prkY)* were detected in the Vi-negative isolates. In addition PCR targeting flanking regions of SPI-10 also yielded negative result. On the other hand all Vi-positive *S*. Typhi isolates yielded positive results under same conditions. WGS analysis of our Vi-negative and Vi-positive strains also confirms absence of SPI-7 and SPI-10.

Unlike SPI-7 which can be either partially or fully lost, SPI-10 is observed as completely absent in Vi-negative strains. It might be because its size is much smaller (33kb). It can be inferred that virulence related genes located on SPIs associated with prophages make up the major differences in gene contents among Vi-negative and Vi-positive *S*. Typhiisolates.

Repetitive DNA including VNTRs in bacterial genomes reflects their genomic diversity. VNTRs are commonly used to differentiate strains of homogenous clones. These are convenient for typing *Salmonella enterica* serovars and many previous studies have reported the VNTR based variability among *S*. Typhi isolates [45,46,47,48,49]. In this study, genetic diversity among studied *S*. Typhi isolates was tested by VNTR based molecular typing. All 35 isolates were clustered into five groups. Overall, all Vi-negative isolates were placed in a single group (T5) whereas Vi-positive isolated were grouped into four types. Both Vi-negative and Vi-positive isolates did not fall into same VNTR type. Unlike our isolates, *S*. Typhi strains from other countries including Nepal [45,46], China [48], Indonesia, Bangladesh, India, Singapore and Malaysia [45]showed more variety in their VNTR types. Octavia and Lan also searched VNTR profiles among *S*. Typhi strains collected from many countries except Pakistan. They also found variety of VNTR types among these global isolates [47]. Overall results of our study showed that Vi-negative and Vi-positive isolates were mutually exclusive in VNTR typing. Unlike *S*. Typhi strains from other counties, isolates from Pakistani showed less variety in their VNTR types.

*S*. Typhi clinical isolates from Pakistan showed genetic homogeneity in most of the virulence genes. They were found to be different only with regard to that of SPI-7 and SPI-10 regions. Our Vi-negative strains were deficient in both SPI-7 and SPI-10, so are reporting first time their simultaneous absencein *S*. Typhi Vi-negative strains. Interestingly, the absence of SPI-10 was also reported in an Indian strain (P-stx-12) that has intact SPI-7 [22]. Both SPI-7 and SPI-10 are associated with bacteriophages and mobile genetic elements, so this is probably the reason for their less stability.

It is hypothesized that Vi-negative isolates have evolved alternative ways to survive and colonize even without virulence genes associated with SPI-7 and SPI-10. The absence of both SPIs from the genome of Vi-negative strain could provide important functional clues for understanding the virulence and persistence of the pathogen, anticipating the need for extensive future studies focusing on their possible roles in bacterial pathogenesis. Our unique finding of SPI-10 deficient Vi-negative variants has further highlighted the importance of naturally occurring Vi-negative *S*. Typhi and thus the need to focus on universally present somatic antigens rather than Vi antigen for the preparation of successful vaccines effective against all isolates of *S*. Typhi.

## Materials and methods

### *S*. Typhi isolates

A total of 35 of *S*. Typhi isolates (Vi-negative (n = 4) and Vi-positive (n = 31)) were included in this study. All isolates were revived from National Institute for Biotechnology and Genetic Engineering (NIBGE, Pakistan) stock cultures previously collected different patients suffering from typhoid fever in various hospitals in Faisalabad region of Pakistan between 2002 and 2006 and stored in 20% glycerol at -20°C.

### Identification and confirmation of isolates

Identification of isolates was performed by conventional biochemical methods after growing them on TSI medium (Merck, Germany). The strains were tested by agglutination for the presence of Vi antigen using Vi monovalent antisera (Monovalent Vi, Bio-Rad, France). These isolates were confirmed by regular and nested polymerase chain reaction (PCR) targeting *fliC-d*, *tviA*, and *tviB* genes as previously reported [50].

### PCR virulotyping

For virulotyping analysis, all 35 *S*. Typhi isolates were screened for the presence/absence of 56 reported virulence genes (Table 3). Virulence determinants were categorized according to their locations and function. Virulence genes located on chromosome and associated with 15 SPIs (SPI-1 to 12, SPI-16 to 18) and other virulence genes not associated with any SPI were studied. These genes are responsible for encoding Type I and III secretion systems, invasions, adhesions, motility, sigma factor, virulence transcriptional regulatory protein, outer membrane protein, peptidase, phosphatase, fimbriae, pili, toxin and purine ribonucleoside efflux pump (Table 3). Most of the genes were detected by PCR using previously reported primer sequences whereas we designed new oligonucleotide primers for some of the virulence genes not studied previously by PCR (Table 3).

**Table 3:** List of primers sequences used to detect the virulence genes of *Salmonella enteric* serovar Typhi isolates.

Sr.#	Location	Gene	Primer Sequence 5`→3`	Annealing Temp°C	Amplicon size (bp)	Reference (Coordinates^a^)
1	SPI-1	*invA* *(STY3019)*	GTGAAATTATCGCCACGTTCGGGCAA TCATCGCACCGTCAAAGGAACC	64	284	[51]
2	SPI-1	*prig* *(STY2994)*	CAGGTAACAGAGGCGCTGGATAAA TTACCGTGTTCGATTGCGCGTTAC	55	121	[52]
3	SPI-1	*hilA/iagA*	ACGGACAGGGTTATCGGTTTAAT AAAAGGAAGTATCGCCAATGTATGAG	50	92	[53]
4	SPI-1	*sipA/sspA*	GTTAAGTAATGTGCTGGACGGCCT ACCCGATCCACACCAGGTTTATTC	55	100	[52]
5	SPI-1	*prgH*	TCATAATCGCCCCTCGCTAA TCTATGTCGCTGCGCAAAAT	50	70	[53]
6	SPI-2	*spiC*	CCTGGATAATGACTATTGAT AGTTTATGGTGATTGCGTAT	50	301	[54]
7	SPI-2	*sseB*	ATATGGCGATCATGGGAAGCTGGA TCGGTATTCCGGTTGGCGTCATTA	55	84	[52]
8	SPI-3	*mgtB*	GGCAGGAGTTTCGCACTAAC GCGTACCCACAATGGATTTC	55	445	[55]
*9*	SPI-3	*mgtC*	TCGGCGTGTTATGCGGCTTA AGCCCTGTTCCTGAGCGGGG	55	264	[56]
10	SPI-3	*nepI/gaiA* (STY4008)	GTTGGCGCTGGGCGGATTCT CACCGGCACCAACGCAAACG	60	616	This study (3870217–3871410)
11	SPI-4	*spi4D* *(STY4457)*	GTTCATGGTCAGGGCGTTAT CTTAAAGAACGGGTGCCATC	55	275	[55]
12	SPI-4	*orfL* *(STY4458)*	GGAGTATCGATAAAGATGTT GCGCGTAACGTCAGAATCAA	50	332	[57]
13	SPI-5	*pipB*	TAAGAAGAAGCAATGAAAGATGGTT GGTTATAAGTGAATCAGGCTGTTGT	50	305	[58]
14	SPI-5	*pipD*	CGGCGATTCATGACTTTGAT CGTTATCATTCGGATCGTAA	50	399	[54]
15	SPI-5	*sopB/sigD*	CGGACCGCCCAGCAACAAAACAAGAAGAAG TAGTGATGCCCGTTATGCGTCAGTGTATT	55	220	[54]
16	SPI-6	*tcf*	CATTTATTCTCAGGGGGAGCG CATCCTCTTTATCTGTTGCCACG	57	1049	[59]
17	SPI-6	*safC*	TGTTCTGGCTCCTTGTTTGACG TTCTGTTTGACCTCCACCCGAG	57		[59]
18	SPI-7	*pilS*	GTATCAACATTAAATCCATGC CGTTACTTTCGCATCGGTGTG	50	502	[18]
19	SPI-7	*tviA*	GTTATTTCAGCATAAGGAG ACTTGTCCGTGTTTTACTC	50	599	[60]
20	SPI-7	*tviB*	CGAGTGAAACCGTTGGTACA CAATGATCGCATCGTAGTGG	50	846	[28]
21	SPI-7	*tviD-E*	TACCTAGCGAGCCAGTACAGAG CTGGAACCGTCATTCTTATCCCG	55	2500	[61]
22	SPI-7	*sopE*	GCTGACTTTGGTGCTGCTGCTCTCG CTGGCGTATGCGGGGTCTTTACTCG	50	2000/2425	[35]
23	SPI-8	STY3280	ATATGACTCGAATGAAATCAGG GGGGATTGTCTACATTGTAA	50	132	[62]
24	SPI-8	STY3282	AAAAAGAGGTCGAGCGCCTTACTCC TTTTAGGAGTGTTTATCATA	50	142	[62]
25	SPI-9	*prtB* (STY2877)	TAACCTGTGCGGCGTGCTGG GCCGGACAGGCCGTTACCAC	55	559	This study (2755839–2757995)
26	SPI-9	STY-2875	TGGCGACACTCTGCTTGGCG CCGTTGAGCGTCGGCTGTGT	50	276	This study (2743495–2754369)
27	SPI-10	*sefC*	GAAGAAAACCACAATTACTC CAACTGTTAGTTTGCTCTTT	50	668	[62]
28	SPI-10	*sefB*	AATATTATGGCCTAAGATTGGG GCTCAATATATCCATTTGGA	50	534	[62]
29	SPI-10	*sefR*	TGACATTCCTACGGCATATG TTACCATTAAGAACAAGTCAAAGCC	50	625	[62]
30	SPI-10	*prpZ*	CAATGGTGCGGTGCGAAAGATAAC TTCCCATAAGGGTCCCATAACTCT	57	115	[42]
31	SPI-10	*prk-Y*	AGCCATGACAAATATGCTCGACCG TTTCCATTCAGGACGAAGAGGGCA	57	104	[42]
32	SPI-10	*prk-X*	CGTCATGTCGGTCGCGTCAATAAT TTGTTGAGGTGTTTGGGTACCTCG	55	127	[42]
33	SPI-11	*pagC*	TTTAATGGTTGGGCCAGCCTATCG TTAAATGTCGCCTTTACCGTGCCG	55	87	[63]
34	SPI-11	*msgA*	GCCAGGCGCACGCGAAATCATCC GCGACCAGCCACATATCAGCCTCTTCAAAC	62	189	[64]
35	SPI-11	*pagD*(STY1880a)	TGGTAGTAAAACCCCGCAACCACC TGGGTTTTGCCGTCGGGCAG	60	89	This study (1782539–1782802)
36	SPI-11	*cdtB*	ACAACTGTCGCATCTCGCCCCGTCATT CAATTTGCGTGGGTTCTGTAGGTGCGAGT	58	268	[11]
37	SPI-12	*sspH2* (STY2467)	GGGCTGCACCCGCAGAAGAG AGACCTCCAGCGTCCGCAGT	60	216	This study (c2300203-2297837)
38	SPI-16	*gtrA* (STY0607)	GCATCAGGCGCTGGCGAACT AGCGAAGCGTGGTGGTGCTG	60	104	This study (c609678-609316)
39	SPI-16	*gtrB* (STY0606)	CATGCAACCGGGGATGCGGT AATCGCCGGCAACACGCTCA	60	364	This study (c609319-608393)
40	SPI-17	STY-2629	TGGGACGGGTTTAATTGGCGCA GCCCATTGAAAAGAGCCGCCG	55	251	This study (2462589–42645111)
41	SPI-18	*clay*	GACCTTTGATGAAACCATAAAAGAG GCATCGATATCTTTATTCGCTTG	50	600	[65]
42	SPI-18	*taiA*	ATATCACCGATGCGGTGGGAATC ACTTTCACCATTCCATCTTCCGGC	55	141	[63]
43	Non-SPI	*cgsA*	TGCAAAGCGATGCCCGTAAATC TTAGCGTTCCACTGGTCGATGGTG	55	151	[66]
44	Non-SPI	*staA/yadN*	CTTTAGAAGCATCGGCACGAAC CGCAATGGTTATGGCTATGGG	57	505	[67]
45	Non-SPI	*stgA*	TGCCAGGTTACGCCACAAACC CGCTGTGGTATCAATCGTGC	60	354	[68]
46	Non-SPI	*fimA*	CCTTTCTCCATCGTCCTGAA TGGTGTTATCTGCCTGACCA	58	85	[69]
47	Non-SPI	*fliA* (STY2164)	ACGCCCCAGTTCCTGCTCCA ACCGCTGGTGCGTCACGAAG	60	277	This study (2008974–2009693)
48	Non-SPI	*rpoS* (STY3049)	CCGCACTCGGTTCGTGGTCC GTCGCGCACTGCGTGGAGAT	60	345	This study (2915077–2916069)
49	Non-SPI	*phoP*	ATGCAAAGCCCGACCATGACG GTATCGACCACCACGATGGTT	62	299	[70]
50	Non-SPI	*tolC*	TACCCAGGCGCAAAAAGAGGCTATC CCGCGTTATCCAGGTTGTTGC	60	161	[64]
51	Non-SPI	*SsifA*	TTTGCCGAACGCGCCCCCACACG GTTGCCTTTTCTTGCGCTTTCCACCCATCT	55	449	[54]
52	Non-SPI	*STY1460*	TACCGGGGTGGATGCGCTGA GCGACAGGCCTGCGAACAGT	60	322	This study 1410094–1412058
53	pRST98	*spvB*	ATGTTGATACTAAATGGTTTTTCA CTATGAGTTGAGTACCCTCATGTT	55	1776	[71]
54	pRST98	*spvR*	ATGGATTTCATTAATAAAAAATTA TCAGAAGGTGGACTGTTTCAGTTT	55	894	[71]
55	R27, pHCM1	*trhW*	ACTGGCCAGGTTCCCGCAGA CTGACCGCTGCCAAGACGCT	60	109	This study
56	Non-SPI	*eno*(STY3081)	GCTCCGTCAGGTGCTTCTAC GCGTCTTTGCCAAGAATAGC	60	143	[72]

^a^Genome coordinates of the location of the genes for which primers have been designed in this study based on *S*. *enterica* Typhi CT8 reference genome [26]

PCR primers were designed for this study from *S*. Typhi CT18 reference genome sequence from the NCBI Genbank (Accession number AL513382) [26]. Primer-BLAST [29] was used for designing the gene specific primers. Each 50μL of reaction mixture for each of the PCR included, 10μL of template DNA, contained 1.5mM MgCl2, 50nmol of each dNTP, 40pM of each (forward and reverse) primer and 2U of *Taq* DNA polymerase (Thermo Scientific, USA). PCR conditions were as follows: initial denaturation at 94°C for 5 min and 30 cycles of denaturation at 94°C for 1 min, annealing at temperature mentioned in Table 1 for each primer set for 1 min and extension at 72°C for 1 min, with a final extension at 72°C for 5 min using T100™ Thermal Cycler (Bio-Rad, USA). A non-template control was included in each run. The PCR products were analyzed by gel electrophoresis on 1.5% agarose stained with ethidium bromide under UV transilluminator.

### Confirmation of absence of SPI-7 and SPI-10 in Vi-negative isolates

Presence of complete SPI-7 was searched by using flanking sides primers [27]. To confirm the complete absence of SPI-10 in Vi-negative isolates, primers specific to flanking sides of SPI-10 were designed (Table 4). The location of these primers on the physical map of the SPI-10 region is shown in Fig 1.

**Table 4:** List of primers used to detect the complete presence/absence of SPI-7 and SPI-10.

Sr.#	Primers	Primer Sequence Forward and Reverse 5`→3`	Annealing Temp°C	Amplicon size (bp)	Reference/(genome Coordinates^a^)
1	DE0032-F DE0083-R	GCTCAGTCGGTAGAGCAGGGGATT TCATCTTCAGGACGGCAGGTAGAATG	57	1275	[27]
2	Spi10up-F Spi10up-R	TTCGAGTCCGGCCTTCGGCA TGCGTCGTGATCCCCCGGAA	50	1134	This study (4683667–4684800)
3	Spi10dn-F Spi10dn-R	CCACCACCCGCGCTCTTTCC CCACAAACCGCTCACCCGGA	50	1039	This study (4716415–4717453)

^a^Genome coordinates of the location of the genes for which primers have been designed in this study based on *S*. *enterica* Typhi CT8 reference genome [26]

#### Whole Genome Sequencing (WGS) analysis

Two Vi-negative and 11 Vi-positive *S*. Typhi strains were analysed by whole genome sequencing to confirm our findings. DNA was isolated by genomic DNA purification kit (**Cat# K0152**, Thermo Scientific, EU) and whole genome paired end sequencing was performed at aScidea Computational Biology Solutions, S.L., Barcelona (Spain) using Illumina HiSeq 2000 system. The sequencing adaptors were removed using built-in Illumina pipeline and raw data was quality checked using FASTQC program and quality filtering and trimming was performed using Trimmomatic v0.35. *de novo* assembly of the short reads was performed using Edena v3 [73] using default parameters. Comparative genome analysis of assembled contigs with *S*. Typhi reference (Accession number: AL513382)was performed using NCBI Blastn [29] and visualized using Circos program [74].

### VNTR based molecular typing

Genetic relatedness of all studied isolates was investigated by Variable Number of Tandem Repeat (VNTR) analysis as described by Liu et al [45]. Three primer pairs, TR1, TR2, and TR3 were used to perform a multiplex PCR (Table 5), and all amplified fragments of different sizes were sequenced. The sequences were then verified by BLASTn[75] against *Salmonella enterica* serovar Typhi. In order to calculate copy number, Tandem Repeat Finder program [76] was used to analyze these sequences.

**Table 5:** List of primers used for *S*. Typhi isolates molecular typing based on VNTR.

Sr. #	Primer	Primer Sequences Forward + Reverse 5`→3`	Repeat sequences	Reference
1	TR1	AGAACCAGCAATGCGCCAACGA CAAGAAGTGCGCATACTACACC	AGAAGAA	[45]
2	TR2	CCCTGTTTTTCGTGCTGATACG CAGAGGATATCGCAACAATCGG	CCAGTTCC	[45]
3	TR3	CGAAGGCGGAAAAAACGTCCTG TGCGATTGGTGTCGTTTCTACC	CGCGGGGATCGGTTTATCCCCGCTGG	[45]

## Supporting information

S1 Table Whole genome sequencing and assembly statisitics of *Salmonella*Typhi Strains. (XLSX)

S1 Fig Comparison of de novo assembled contig lengths between all strains. The x-axis shows the percentage of length of the assembled genome for any strain and y-axis shows the length of contigs in kilobases used for a particular length percentage of assembled genome.
(TIF)

S2 Fig Comparison of de novo assembled contig lengths between all strains with respect to the reference genome size [*S*. Typhi, Acc: AL513382]. The x-axis shows the percentage of length assembled with respect to the reference genome for any strain and y-axis shows the length of contigs in kilobases used for a particular length percentage.
(TIF)

S3 Fig Comparison of growth of contig lengths. On the x-axis, contigs are ordered from the largest to smallest. The y-axis gives the size of the x largest contigs in the assembly.
(TIF)

S4 Fig Comparative distribution of # contigs with GC percentage in a certain range. The x value is the GC percentage intervals. The y value is the number of contigs which GC content lies in the corresponding interval.
(TIF)

S5 Fig %GC content of a ST5 de novo assembled contigs. (TIF)

S6 Fig %GC content of a ST6 de novo assembled contigs. (TIF)

S7 Fig %GC content of a ST7 de novo assembled contigs. (TIF)

S8 Fig %GC content of a ST8 de novo assembled contigs. (TIF)

S9 Fig %GC content of a ST11 de novo assembled contigs. (TIF)

S10 Fig %GC content of a ST16 de novo assembled contigs. (TIF)

S11 Fig %GC content of a ST17 de novo assembled contigs. (TIF)

S12 Fig %GC content of a ST18 de novo assembled contigs. (TIF)

S13 Fig %GC content of a ST19 de novo assembled contigs. (TIF)

S14 Fig %GC content of a ST22 de novo assembled contigs. (TIF)

S15 Fig %GC content of a ST25 de novo assembled contigs. (TIF)

S16 Fig %GC content of a ST26 de novo assembled contigs. (TIF)

S17 Fig %GC content of a ST38 de novo assembled contigs. (TIF)

S18 Fig Distribution of total contig lengths (y-axis) at different read coverage depths (x-axis, grouped in bins). (TIF)

S19 Fig Distribution of contig lengths (y-axis) at different read coverage depths (x-axis) of ST5. (TIF)

S20 Fig Distribution of contig lengths (y-axis) at different read coverage depths (x-axis) of ST6. (TIF)

S21 Fig Distribution of contig lengths (y-axis) at different read coverage depths (x-axis) of ST7. (TIF)

S22 Fig Distribution of contig lengths (y-axis) at different read coverage depths (x-axis) of ST8. (TIF)

S23 Fig Distribution of contig lengths (y-axis) at different read coverage depths (x-axis) of ST11. (TIF)

S24 Fig Distribution of contig lengths (y-axis) at different read coverage depths (x-axis) of ST16. (TIF)

S25 Fig Distribution of contig lengths (y-axis) at different read coverage depths (x-axis) of ST17. (TIF)

S26 Fig Distribution of contig lengths (y-axis) at different read coverage depths (x-axis) of ST18. (TIF)

S27 Fig Distribution of contig lengths (y-axis) at different read coverage depths (x-axis) of ST19. (TIF)

S28 Fig Distribution of contig lengths (y-axis) at different read coverage depths (x-axis) of ST22. (TIF)

S29 Fig Distribution of contig lengths (y-axis) at different read coverage depths (x-axis) of ST25. (TIF)

S30 Fig Distribution of contig lengths (y-axis) at different read coverage depths (x-axis) of ST26. (TIF)

S31 Fig Distribution of contig lengths (y-axis) at different read coverage depths (x-axis) of ST38. (TIF)
