# Supplementary figures and images for: Virulotyping of Salmonella enterica serovar Typhi isolates from Pakistan: Absence of complete SPI-10 in Vi negative isolates
Source: PLoS Negl Trop Dis. 2018 Nov 30;12(11):e0006839. doi: 10.1371/journal.pntd.0006839 (PMC6267989; doi:10.1371/journal.pntd.0006839)

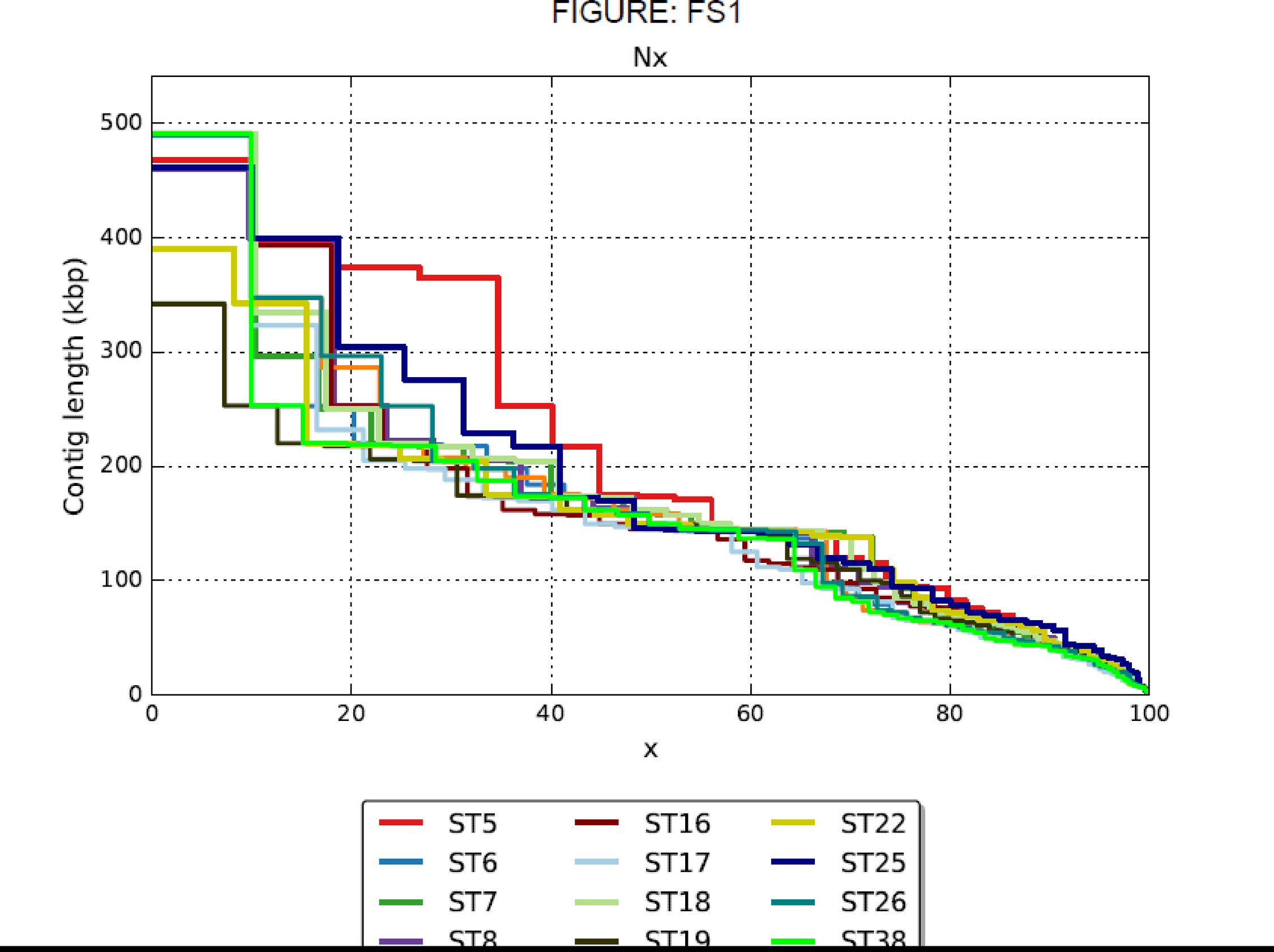

Supplement: S1 Fig — The x-axis shows the percentage of length of the assembled genome for any strain and y-axis shows the length of contigs in kilobases used for a particular length percentage of assembled genome. (TIF) [file pntd.0006839.s002.tif]

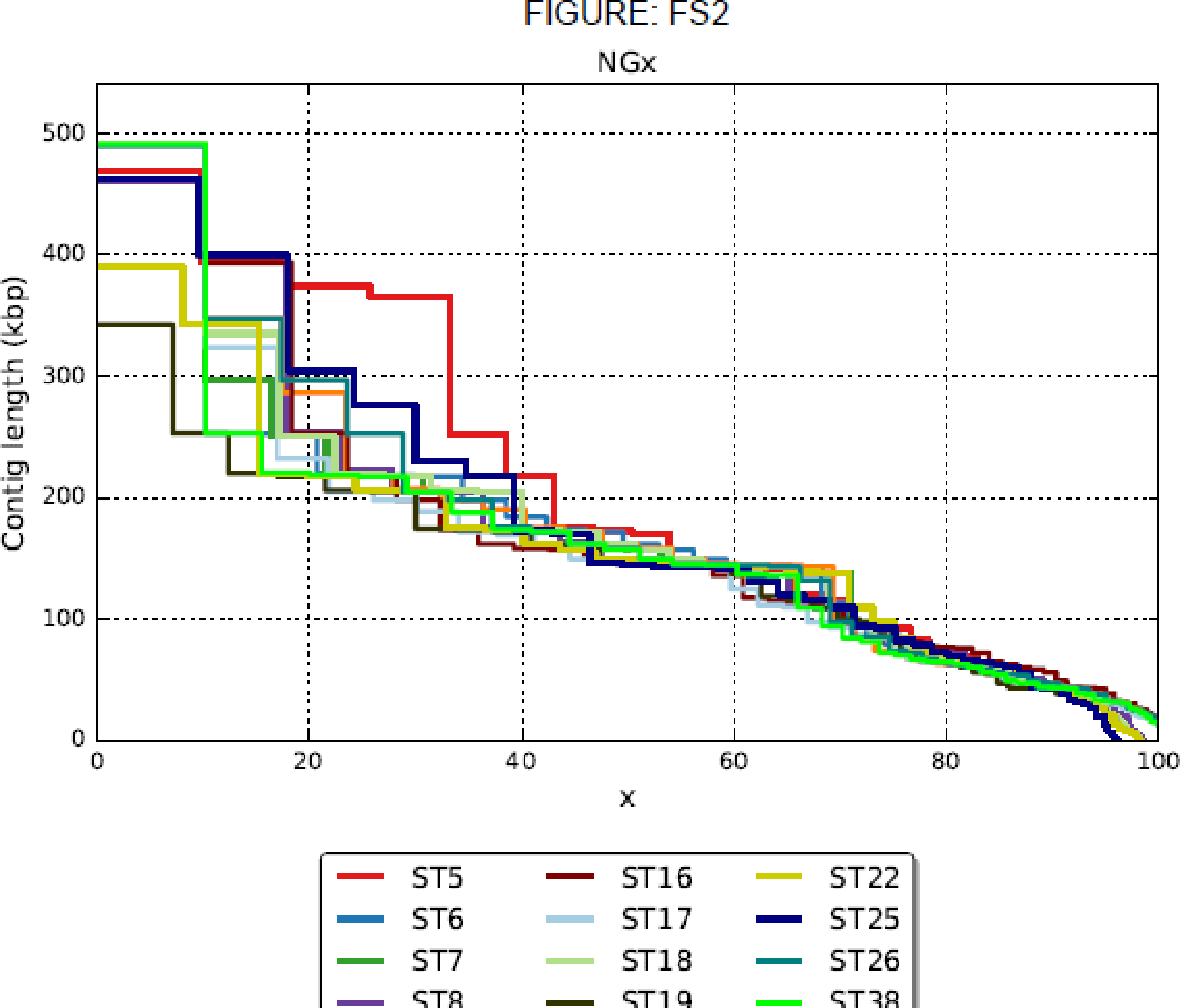

Supplement: S2 Fig — The x-axis shows the percentage of length assembled with respect to the reference genome for any strain and y-axis shows the length of contigs in kilobases used for a particular length percentage. (TIF) [file pntd.0006839.s003.tif]

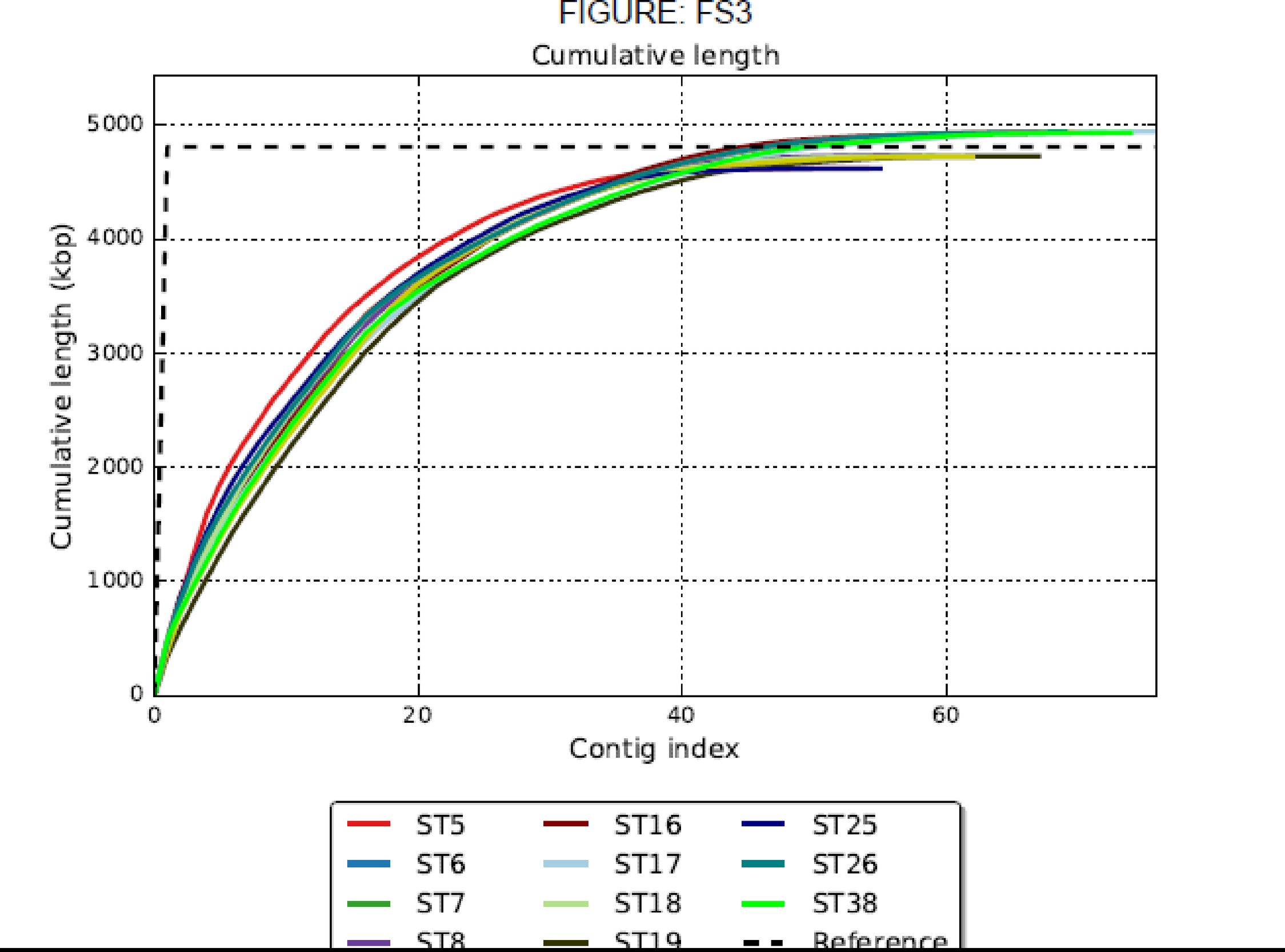

Supplement: S3 Fig — On the x-axis, contigs are ordered from the largest to smallest. The y-axis gives the size of the x largest contigs in the assembly. (TIF) [file pntd.0006839.s004.tif]

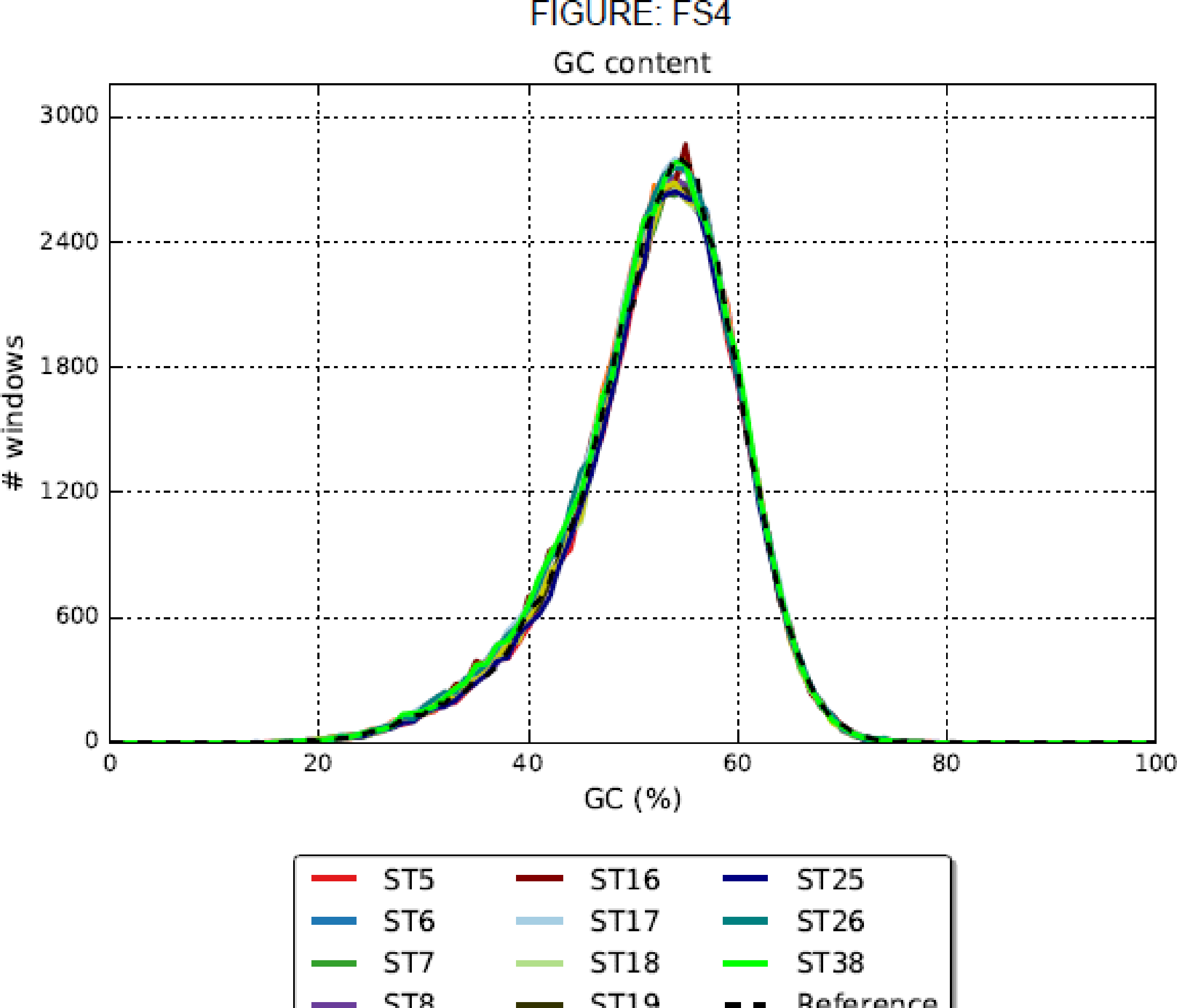

Supplement: S4 Fig — The x value is the GC percentage intervals. The y value is the number of contigs which GC content lies in the corresponding interval. (TIF) [file pntd.0006839.s005.tif]

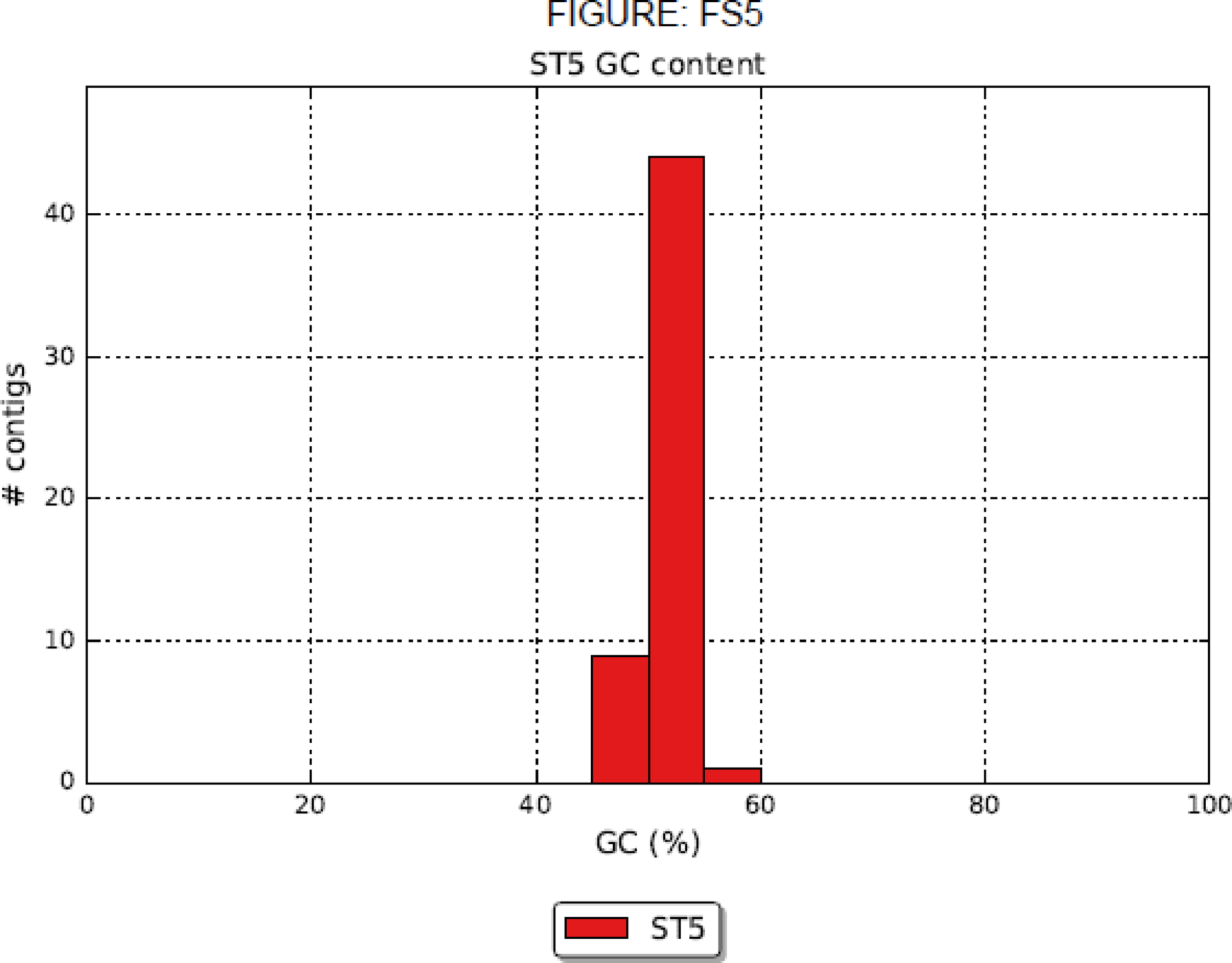

Supplement: S5 Fig — (TIF) [file pntd.0006839.s006.tif]

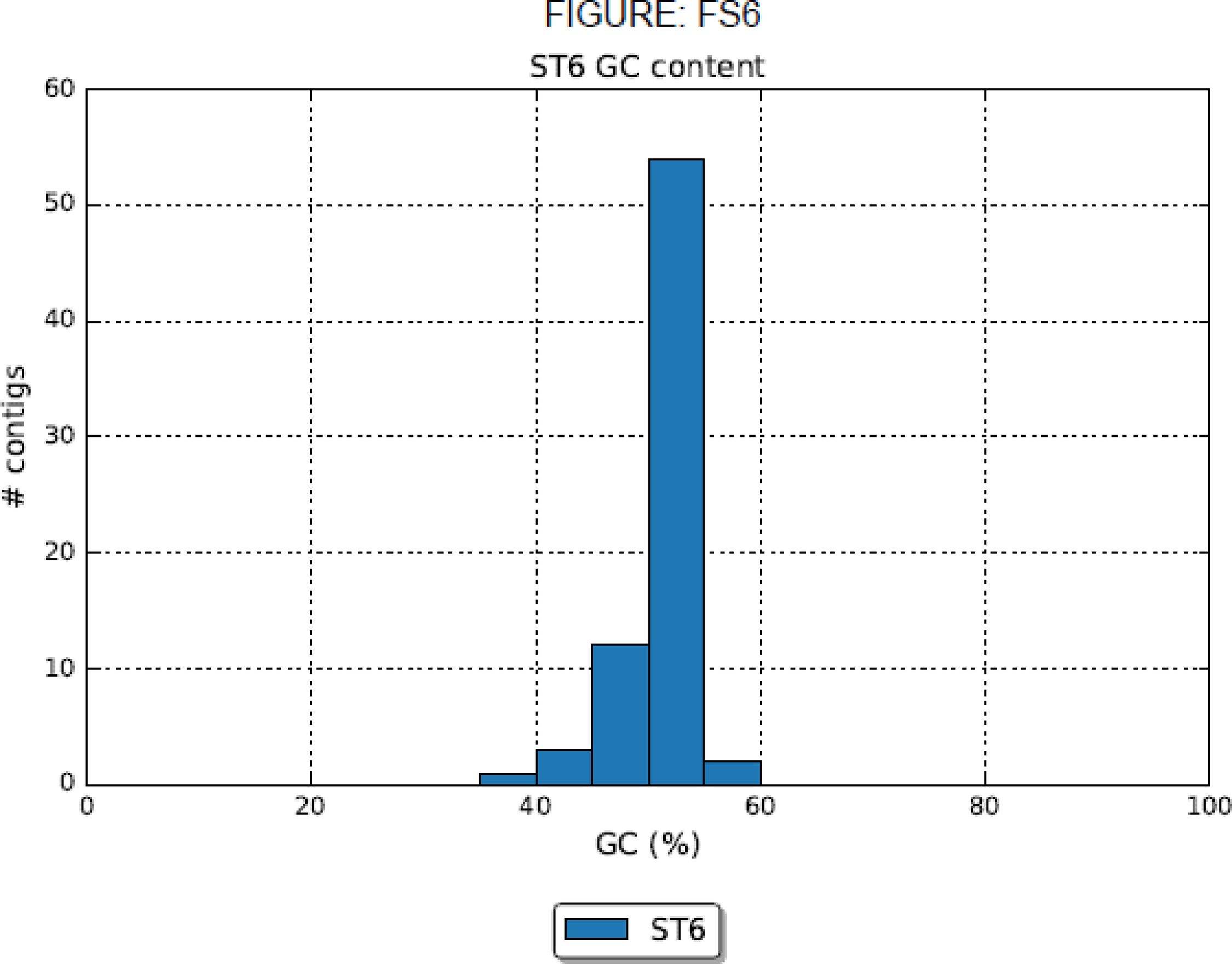

Supplement: S6 Fig — (TIF) [file pntd.0006839.s007.tif]

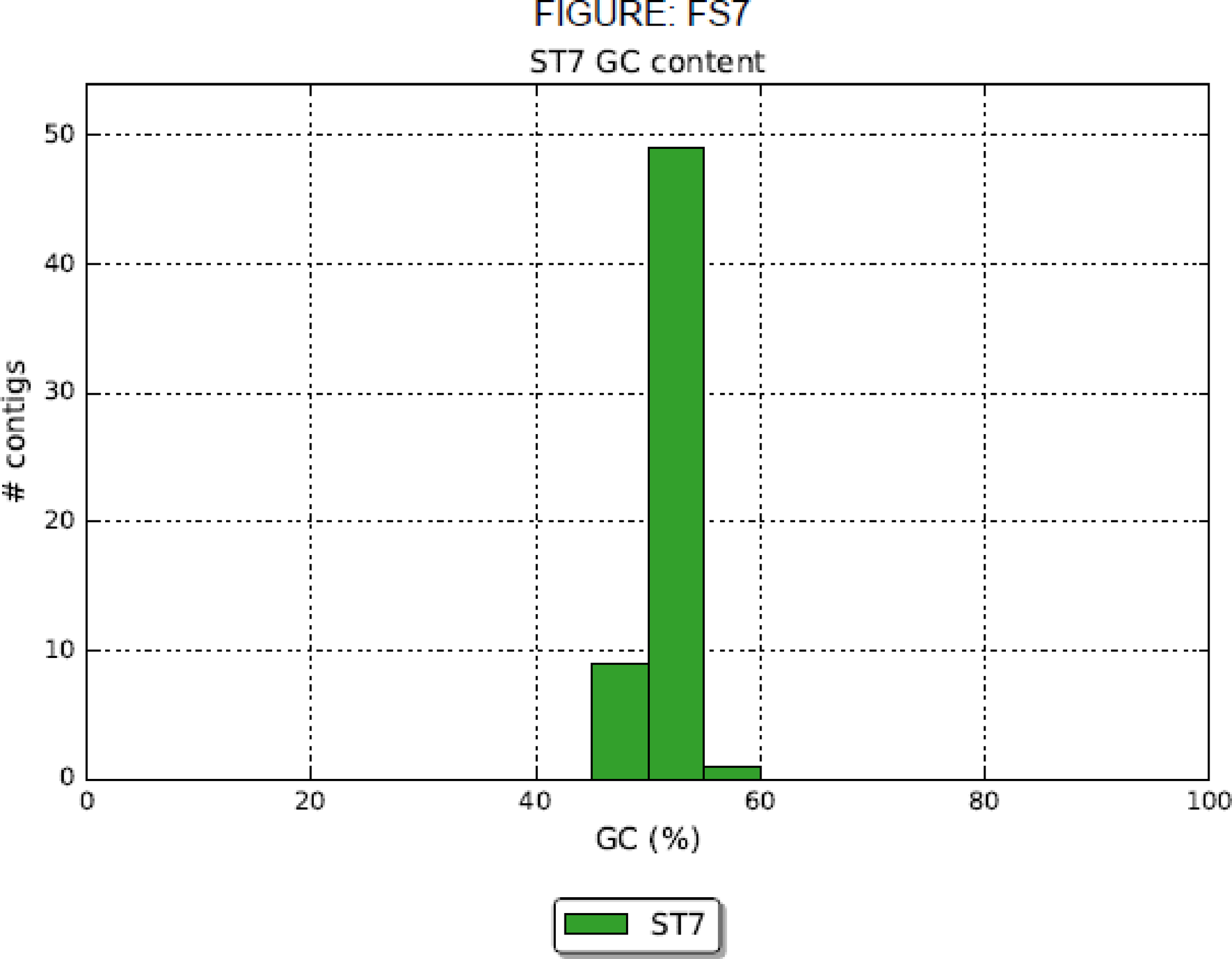

Supplement: S7 Fig — (TIF) [file pntd.0006839.s008.tif]

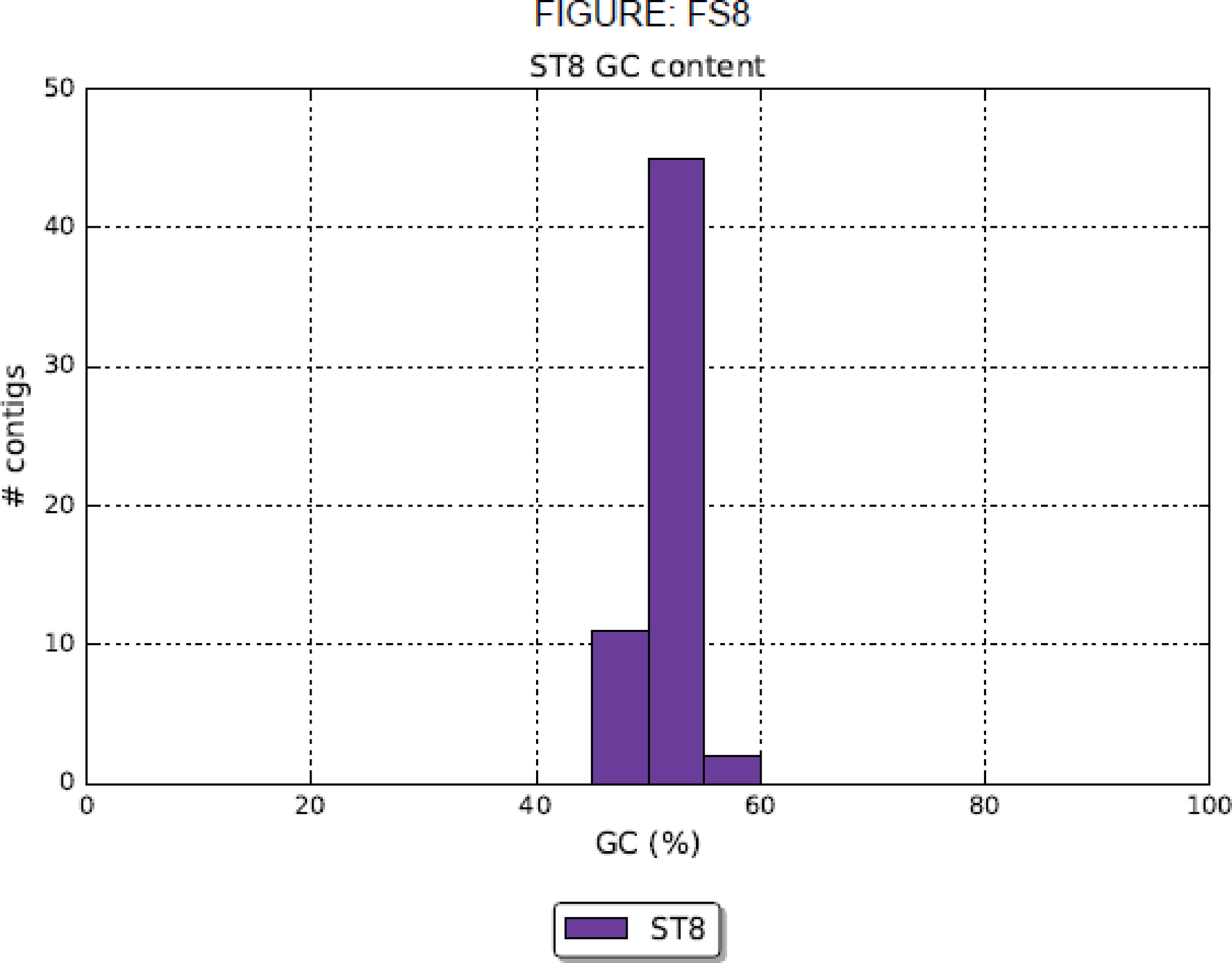

Supplement: S8 Fig — (TIF) [file pntd.0006839.s009.tif]

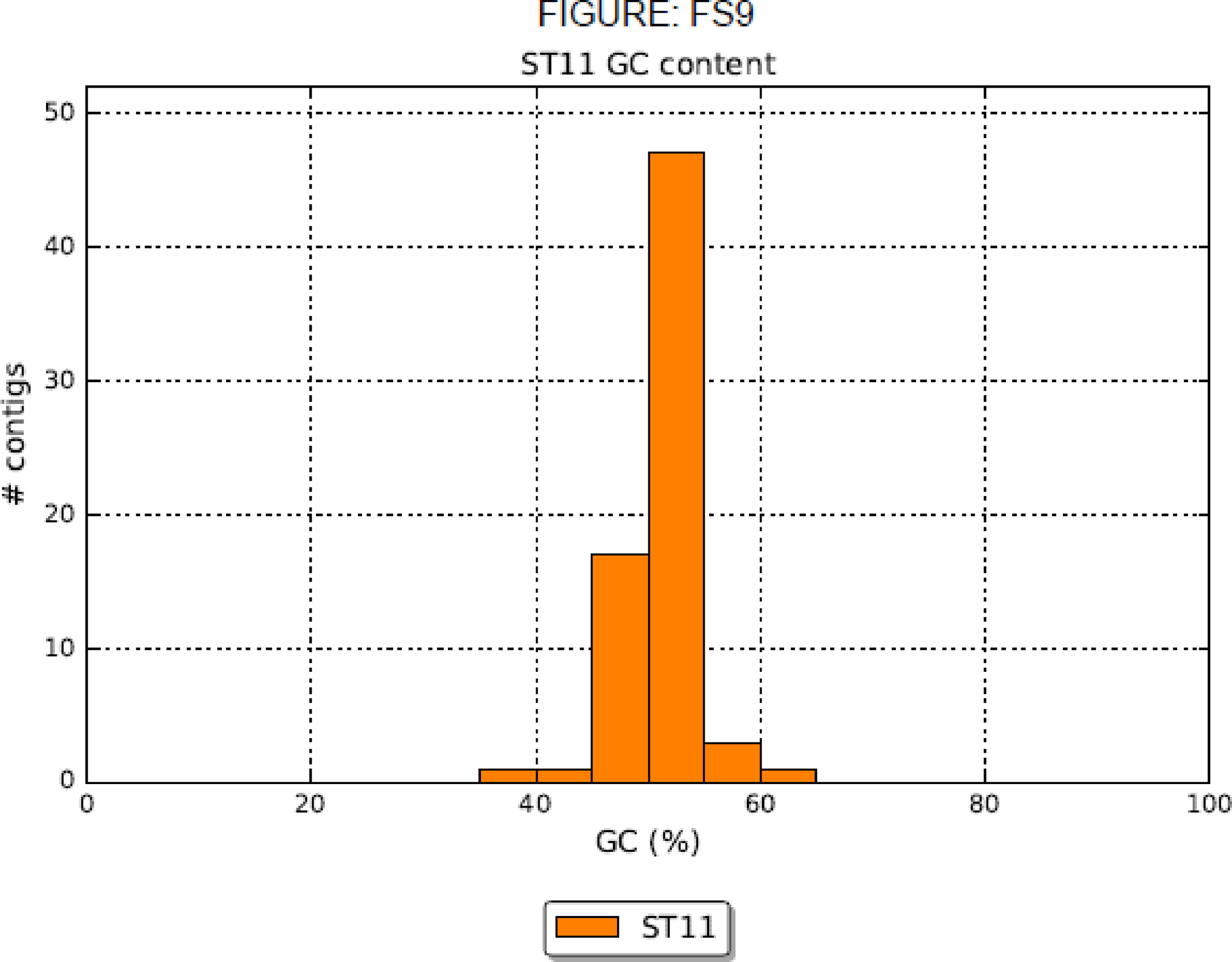

Supplement: S9 Fig — (TIF) [file pntd.0006839.s010.tif]

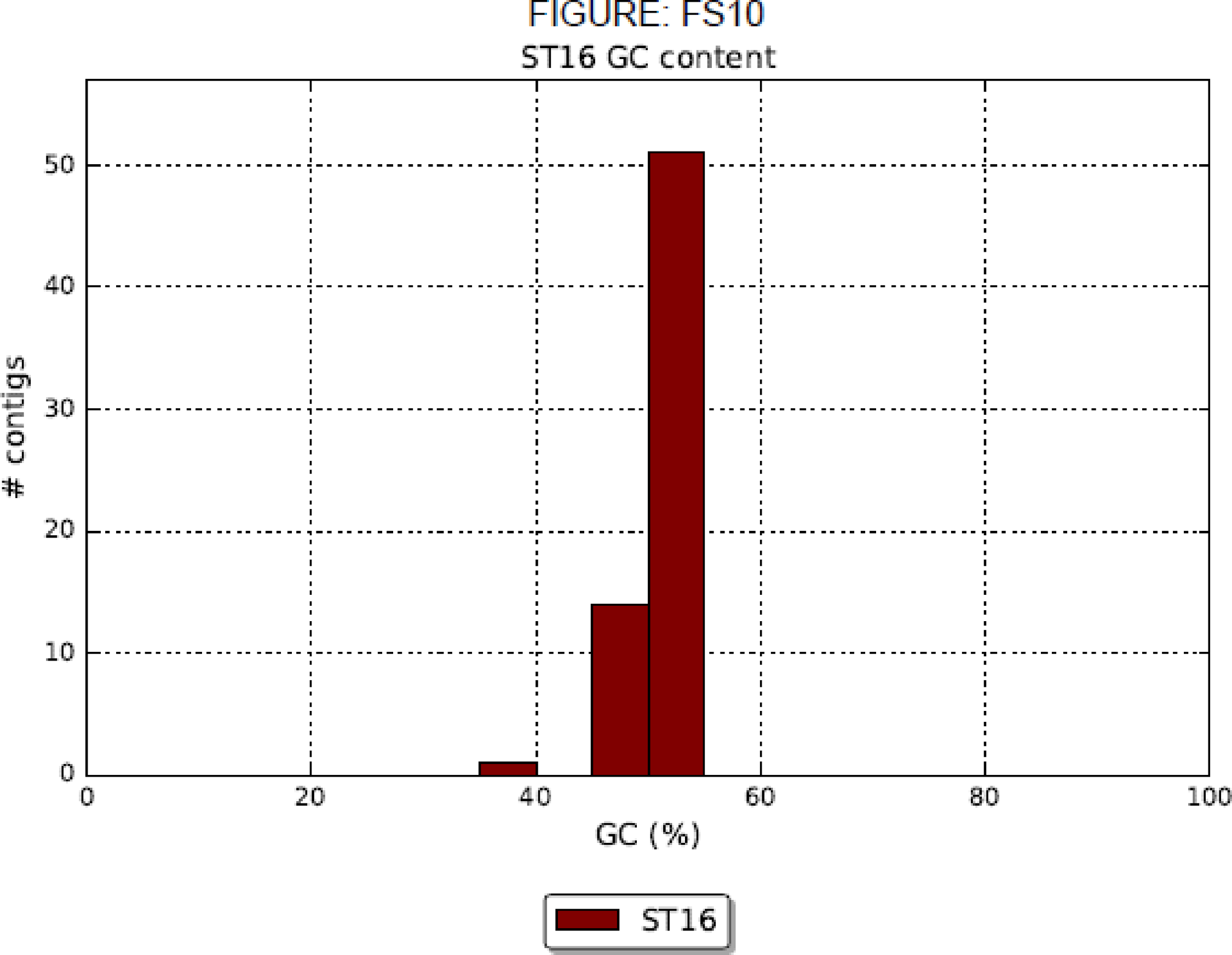

Supplement: S10 Fig — (TIF) [file pntd.0006839.s011.tif]

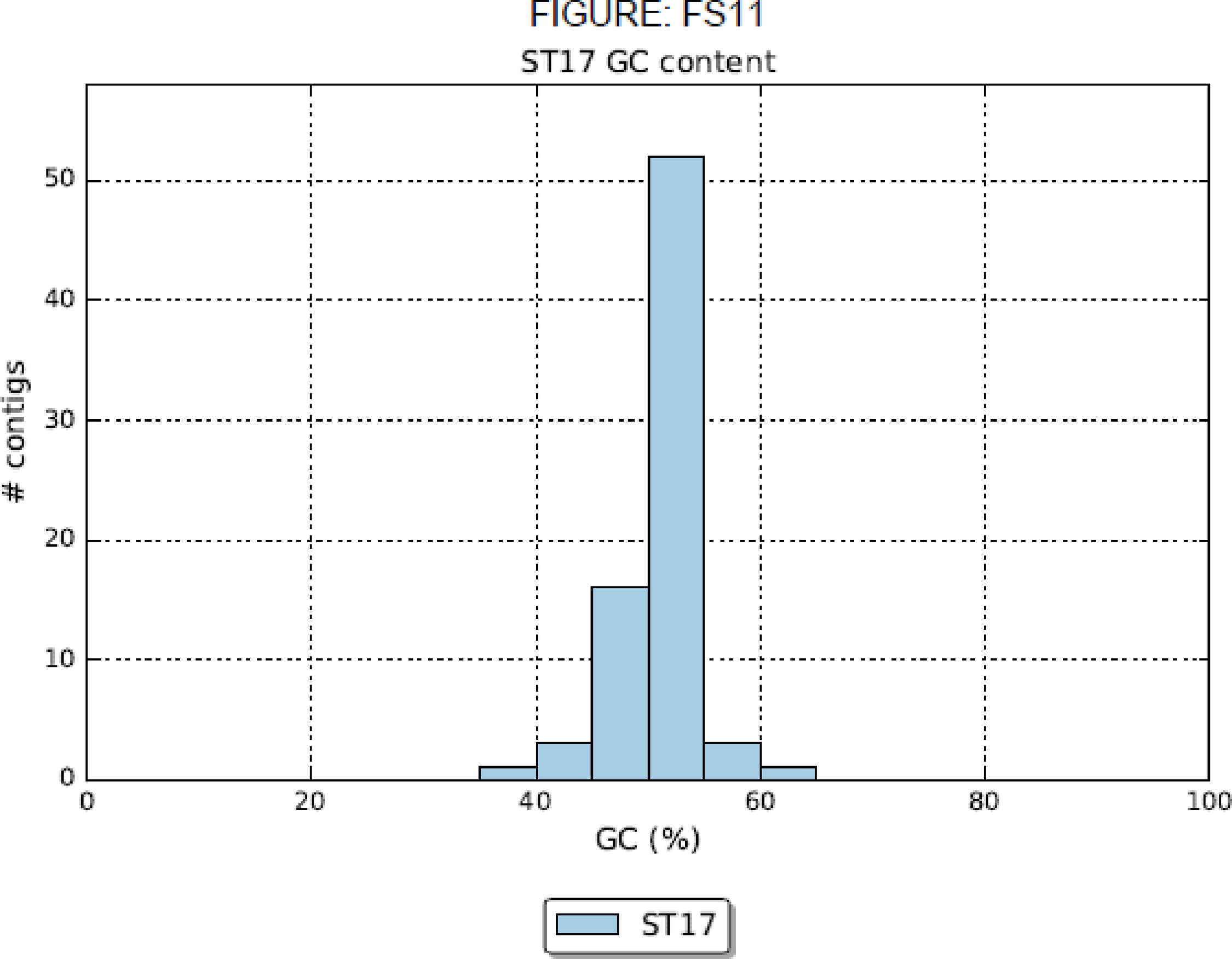

Supplement: S11 Fig — (TIF) [file pntd.0006839.s012.tif]

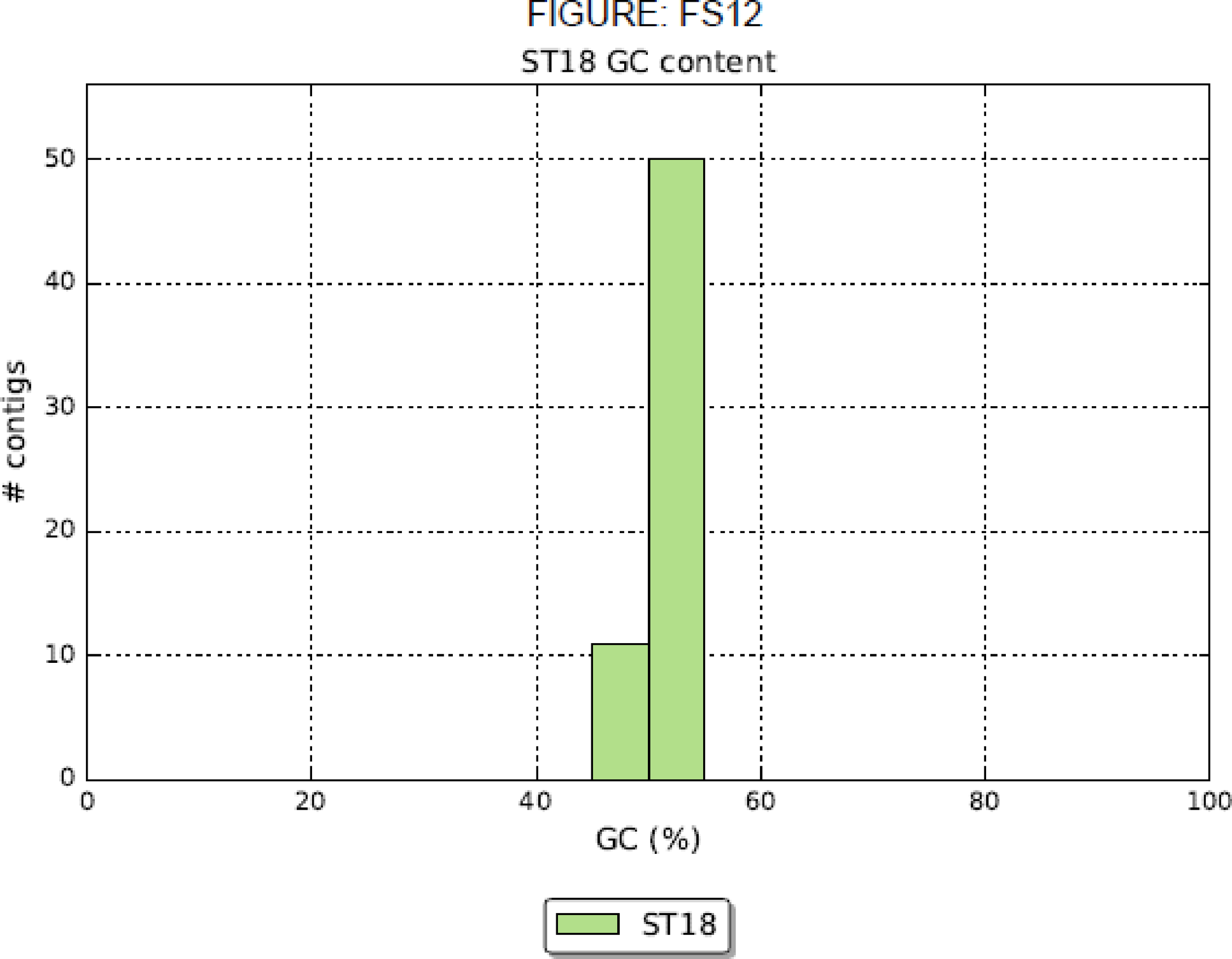

Supplement: S12 Fig — (TIF) [file pntd.0006839.s013.tif]

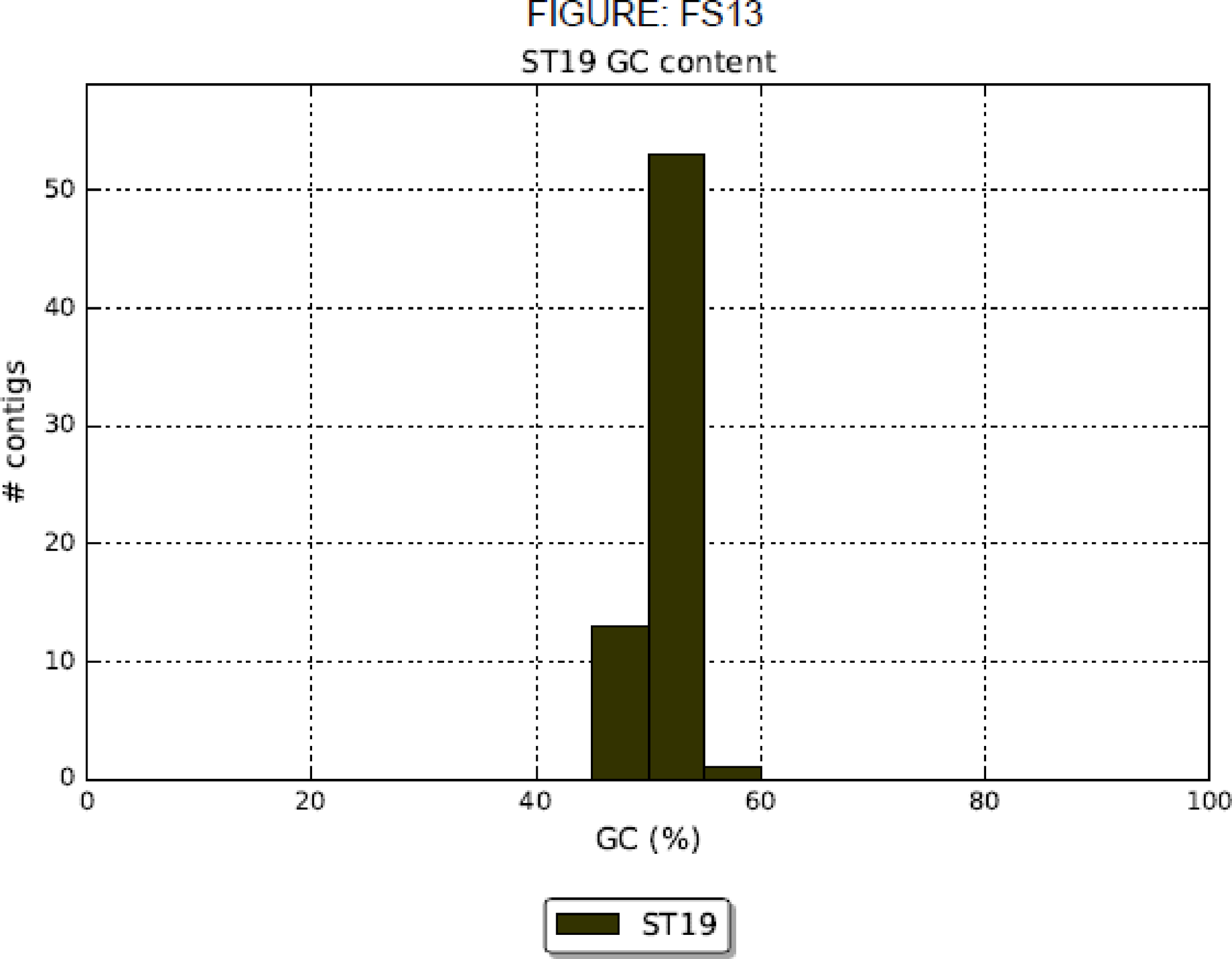

Supplement: S13 Fig — (TIF) [file pntd.0006839.s014.tif]

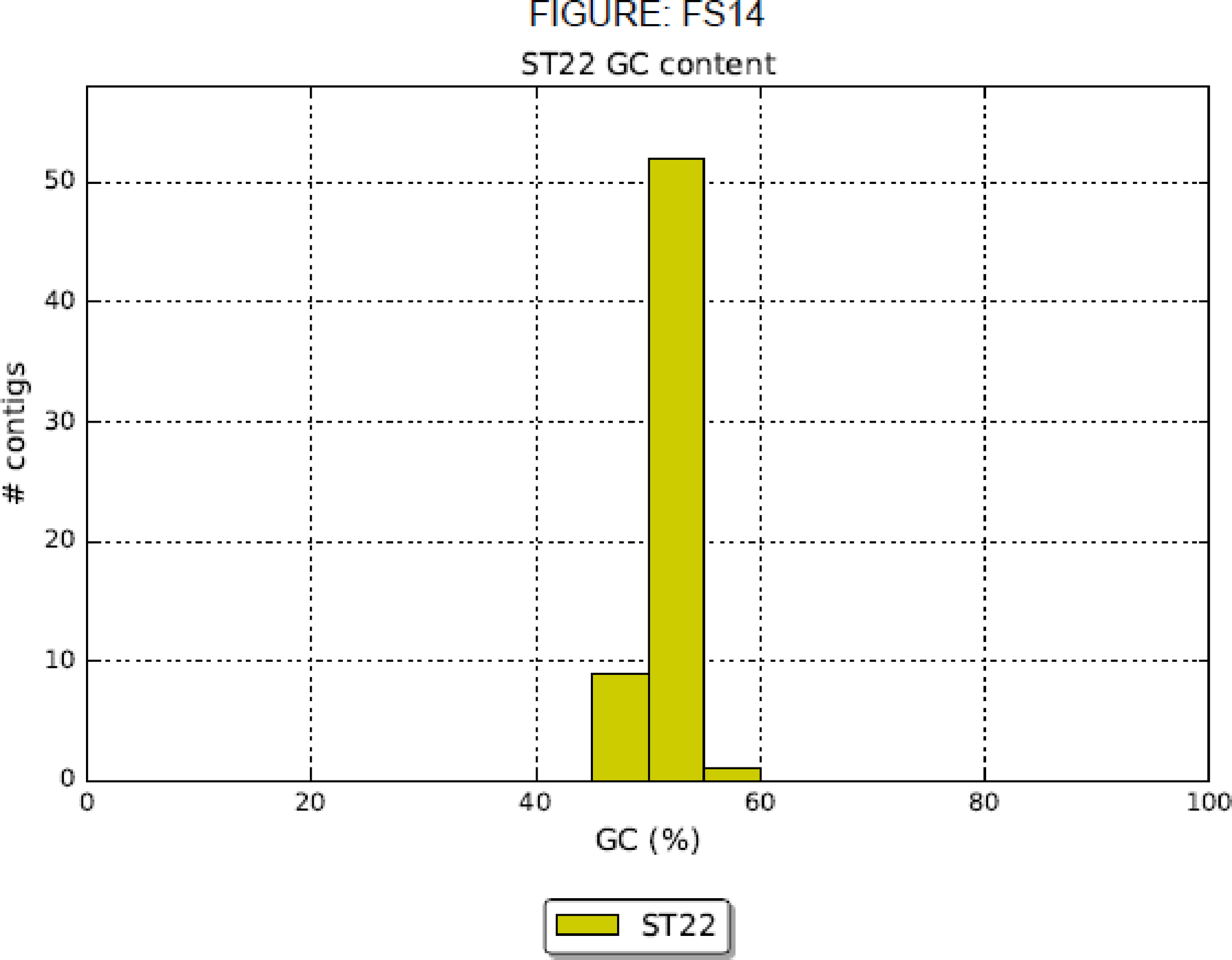

Supplement: S14 Fig — (TIF) [file pntd.0006839.s015.tif]

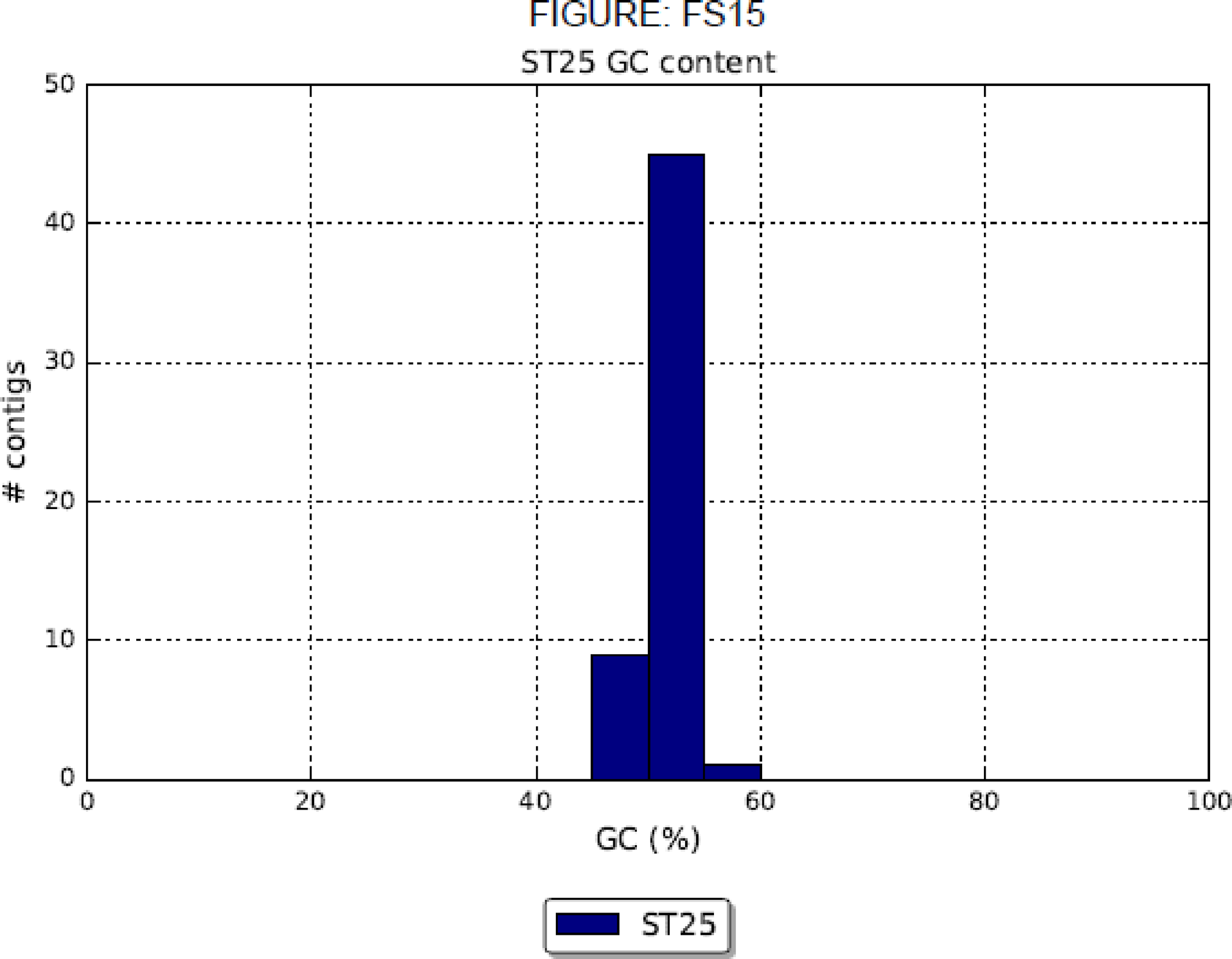

Supplement: S15 Fig — (TIF) [file pntd.0006839.s016.tif]

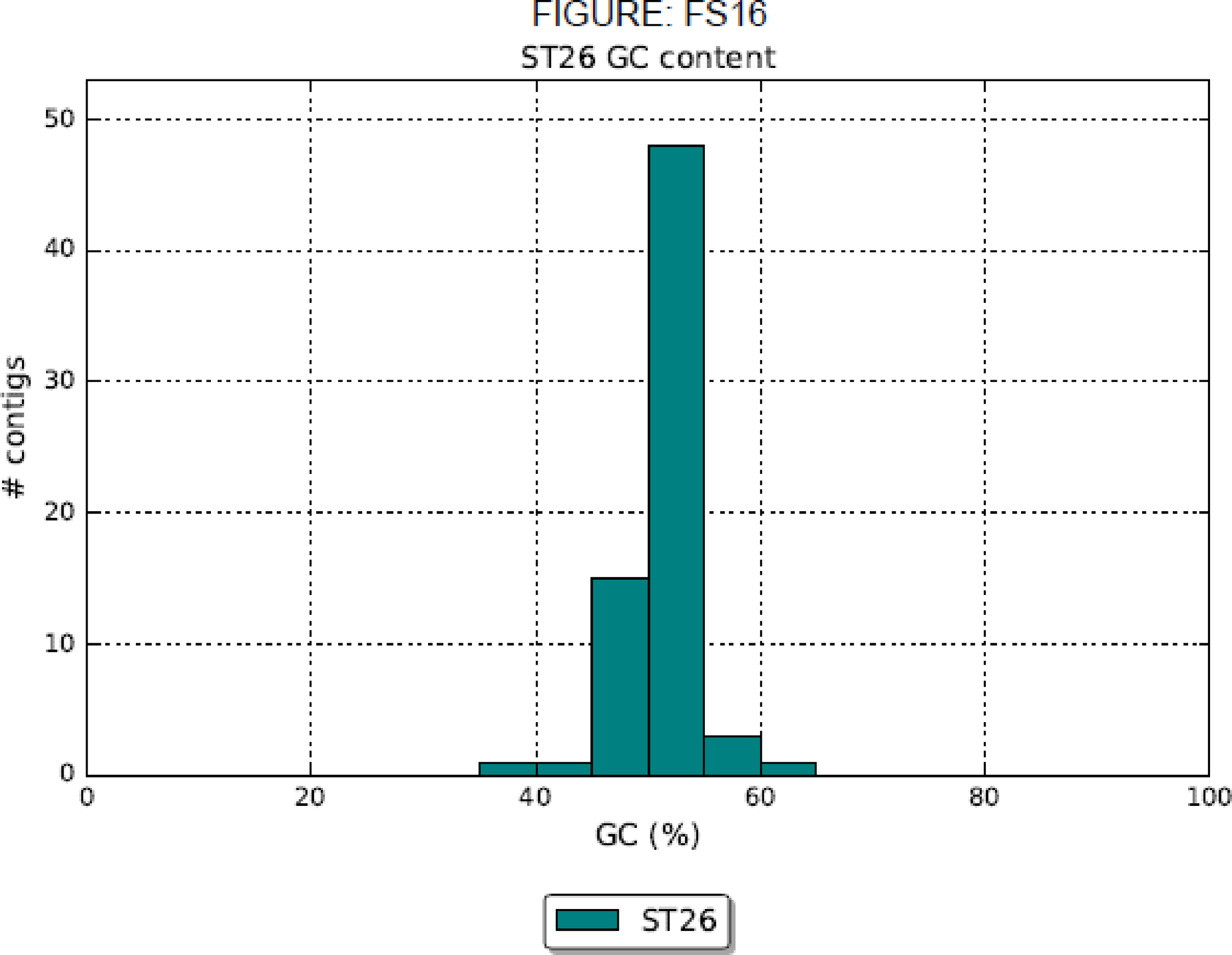

Supplement: S16 Fig — (TIF) [file pntd.0006839.s017.tif]

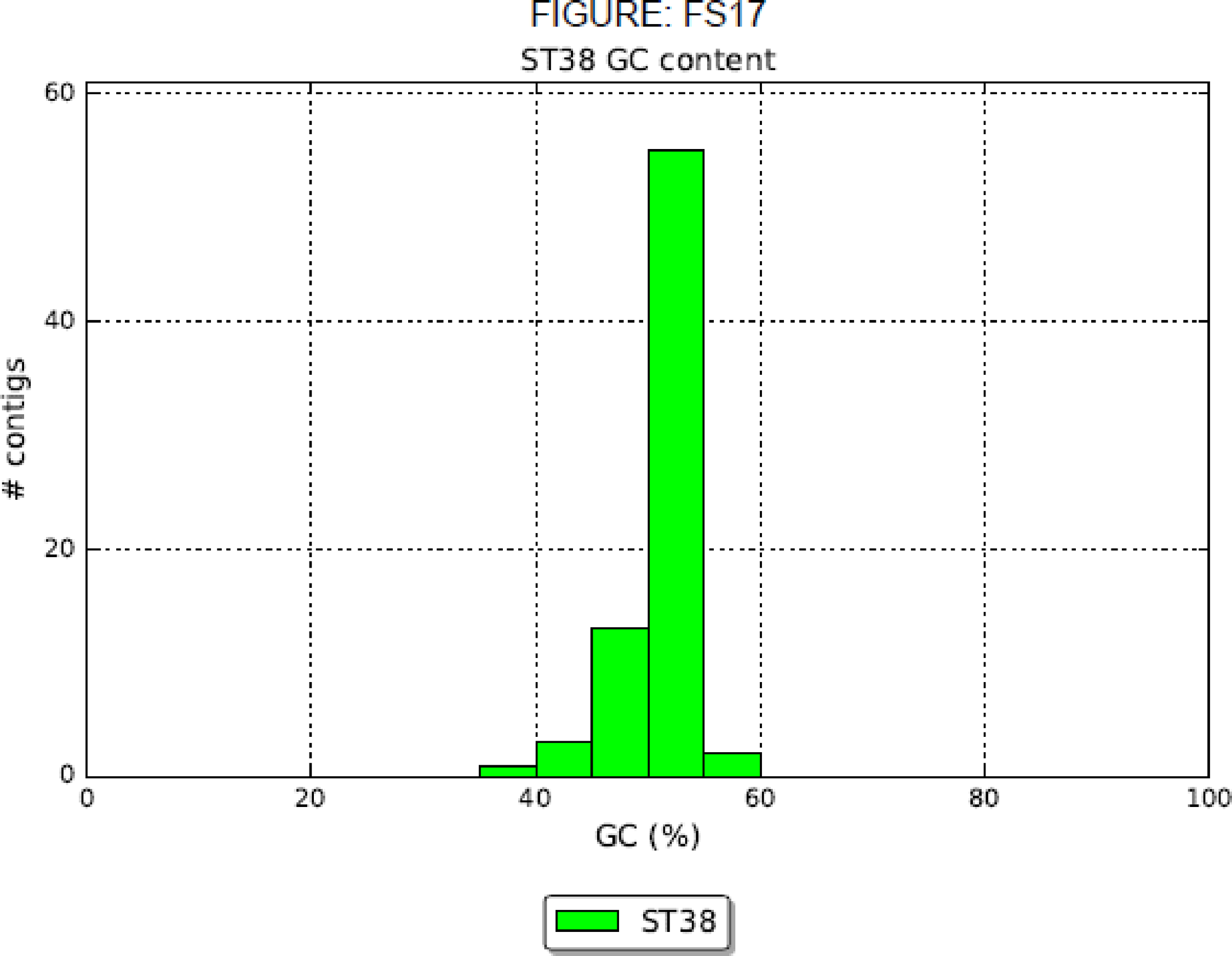

Supplement: S17 Fig — (TIF) [file pntd.0006839.s018.tif]

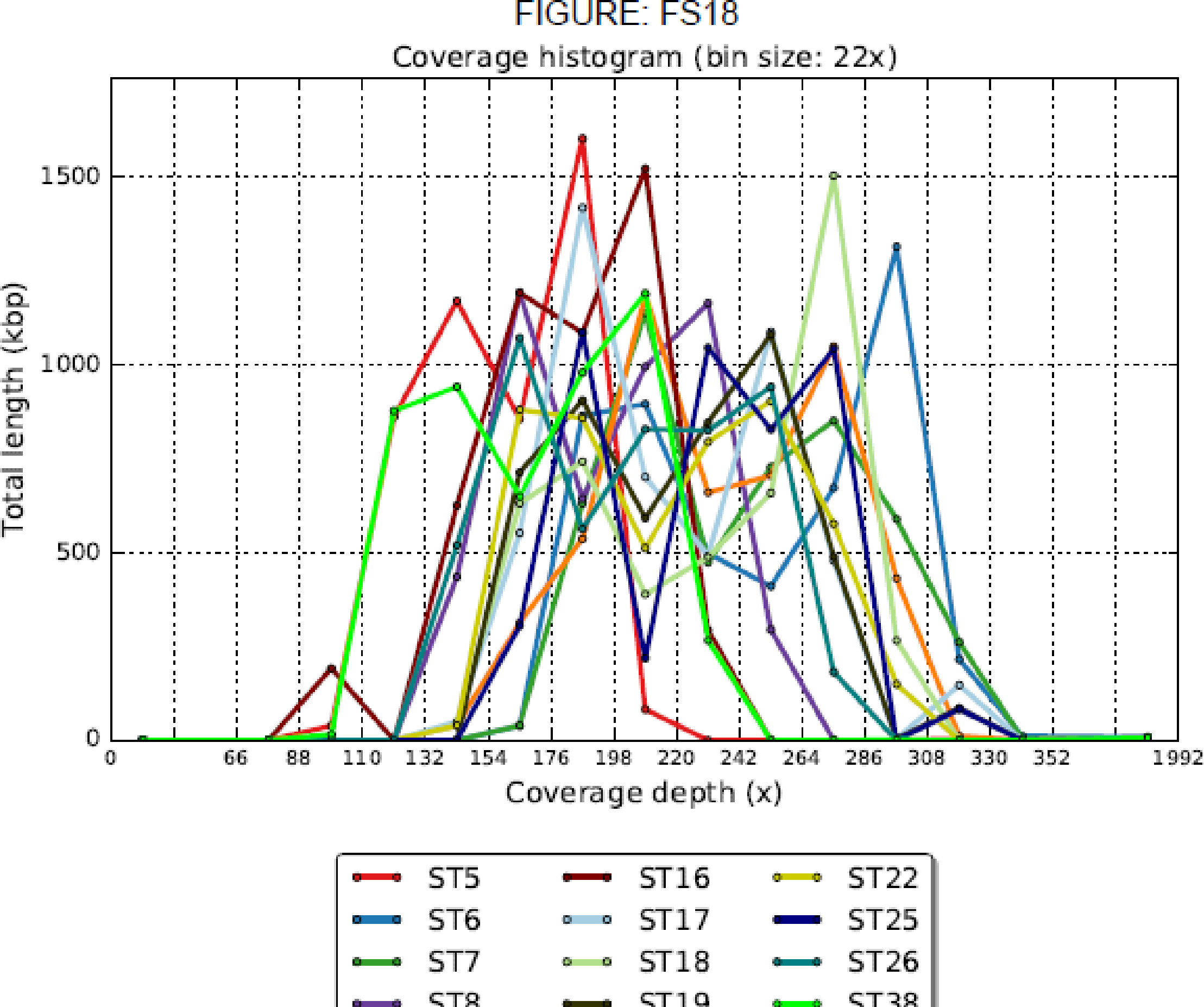

Supplement: S18 Fig — (TIF) [file pntd.0006839.s019.tif]

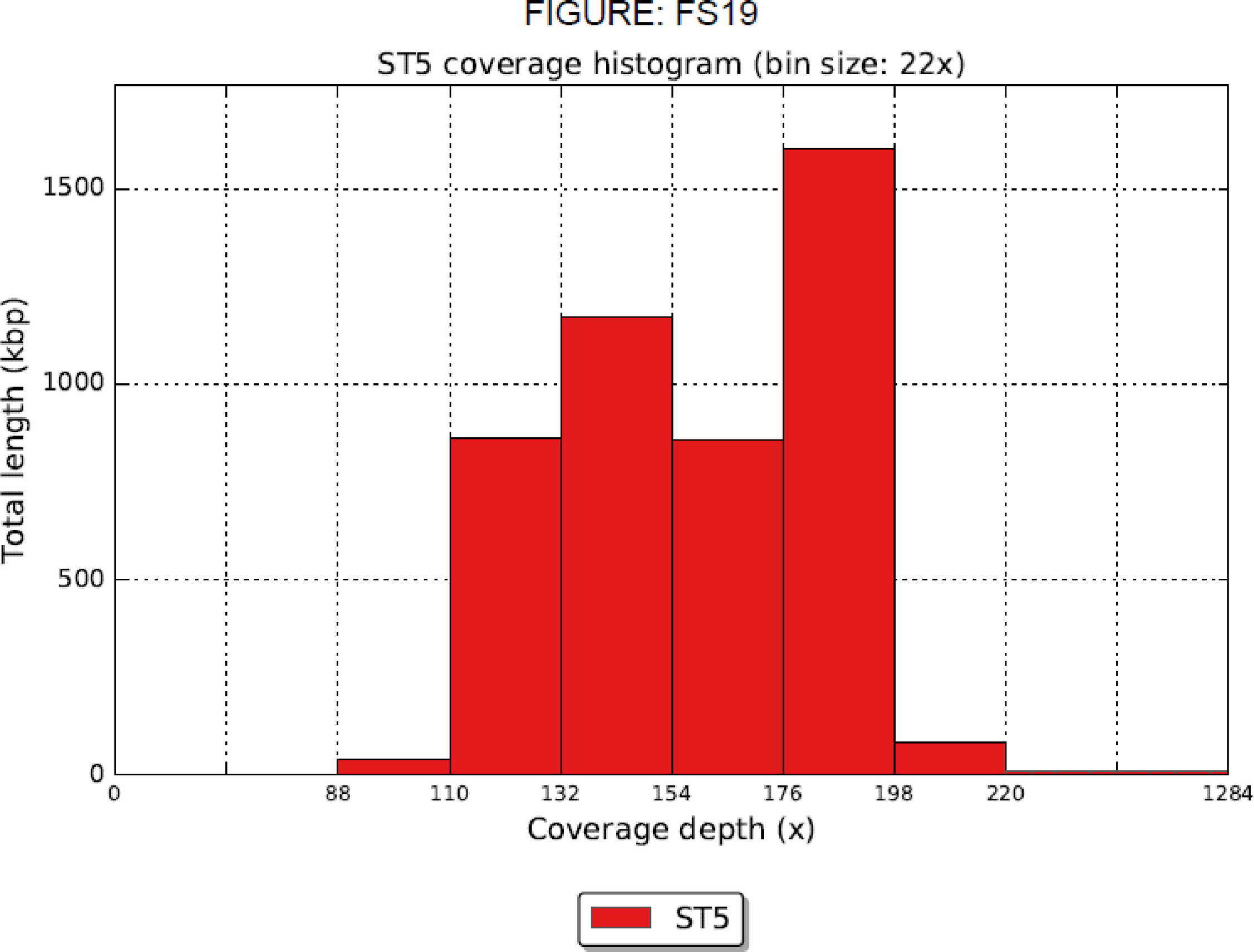

Supplement: S19 Fig — (TIF) [file pntd.0006839.s020.tif]

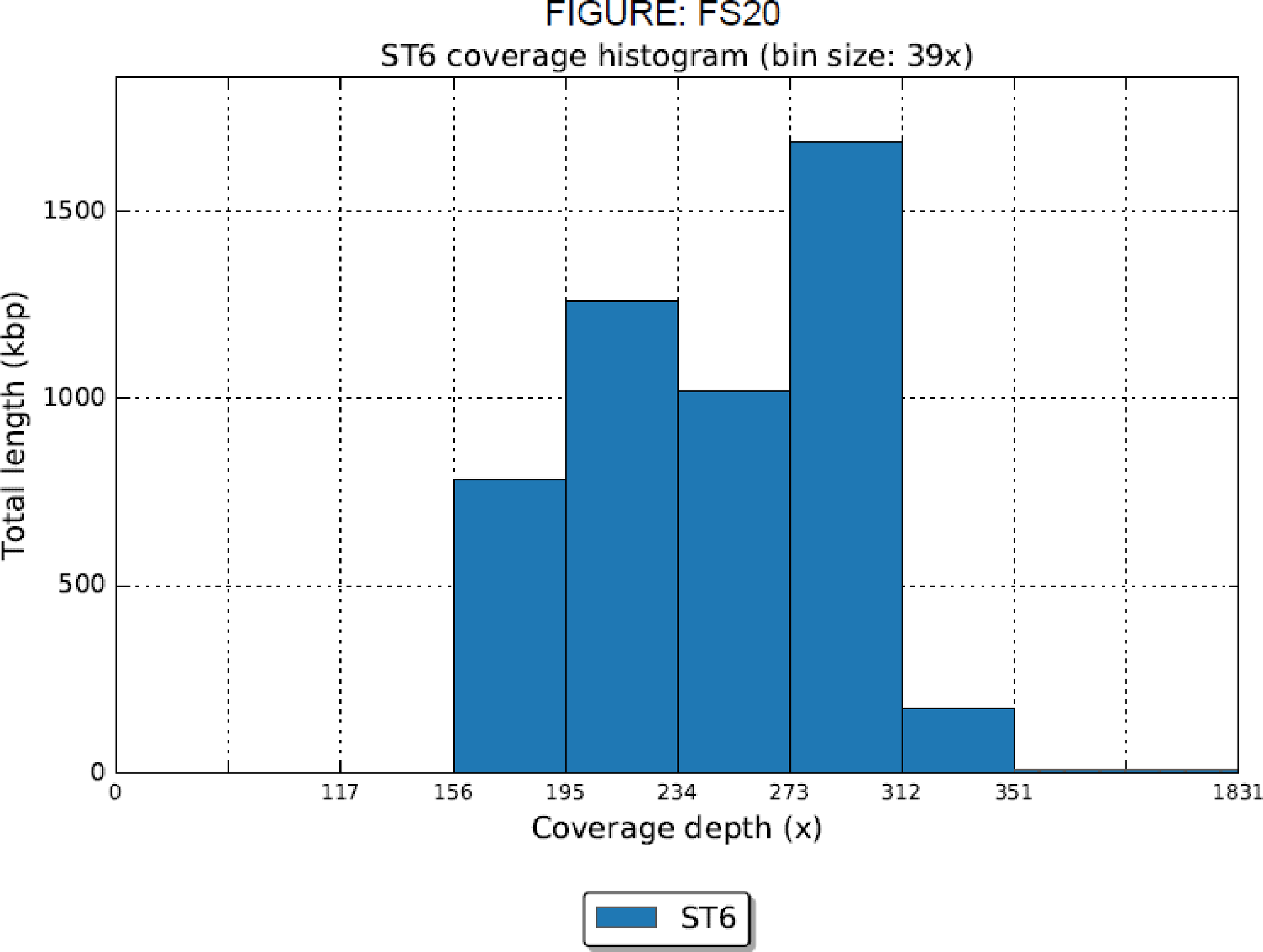

Supplement: S20 Fig — (TIF) [file pntd.0006839.s021.tif]

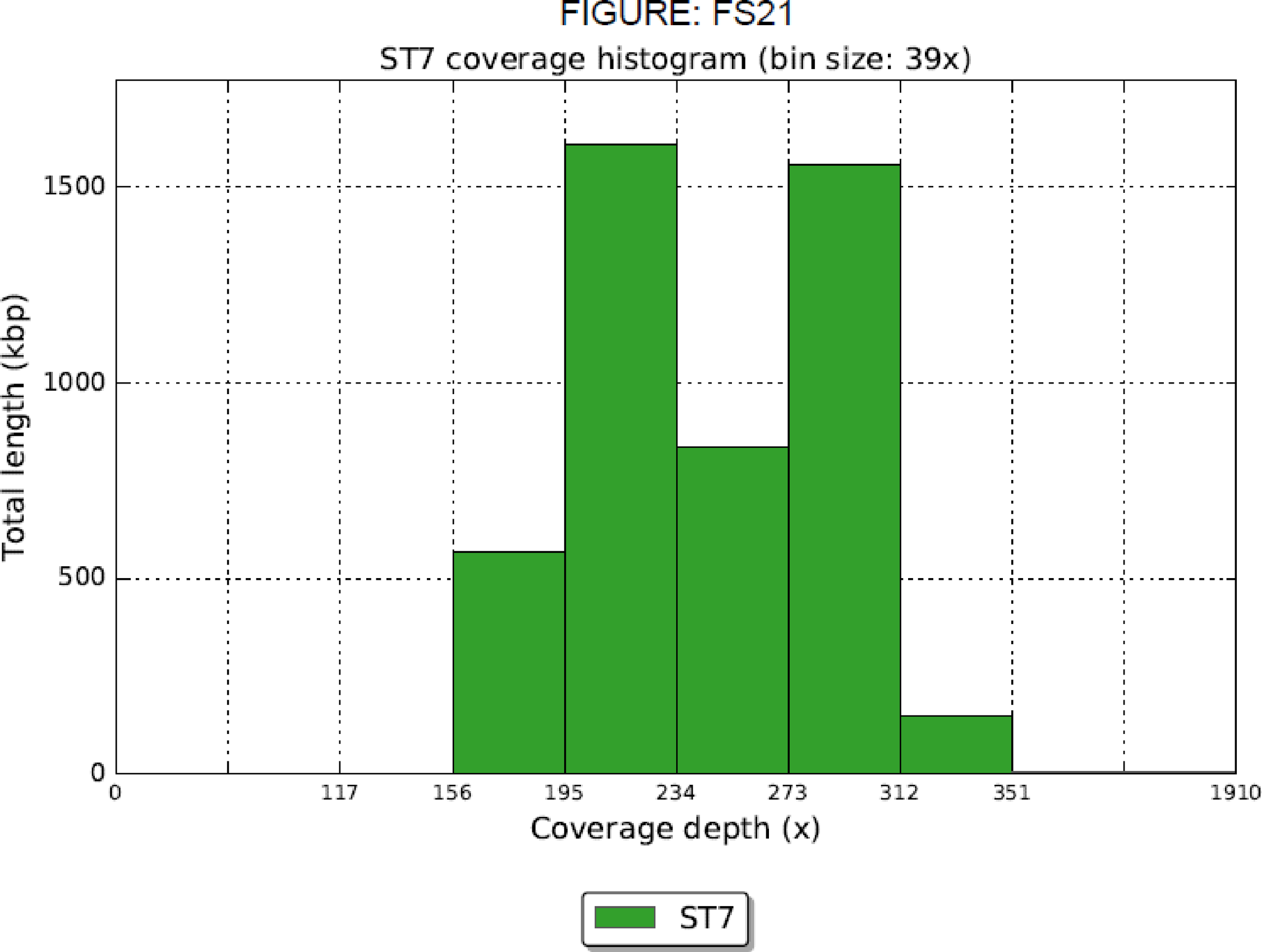

Supplement: S21 Fig — (TIF) [file pntd.0006839.s022.tif]

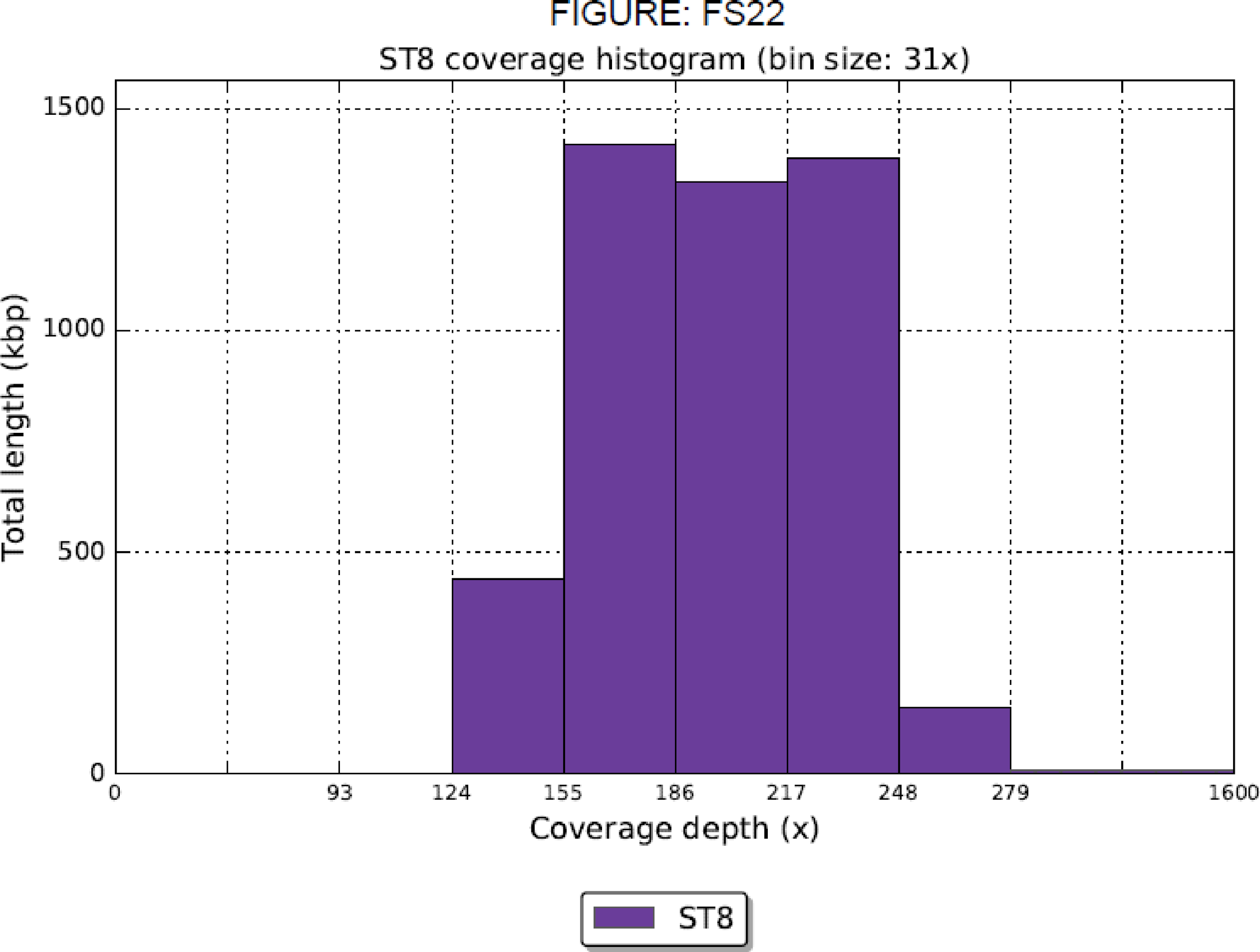

Supplement: S22 Fig — (TIF) [file pntd.0006839.s023.tif]

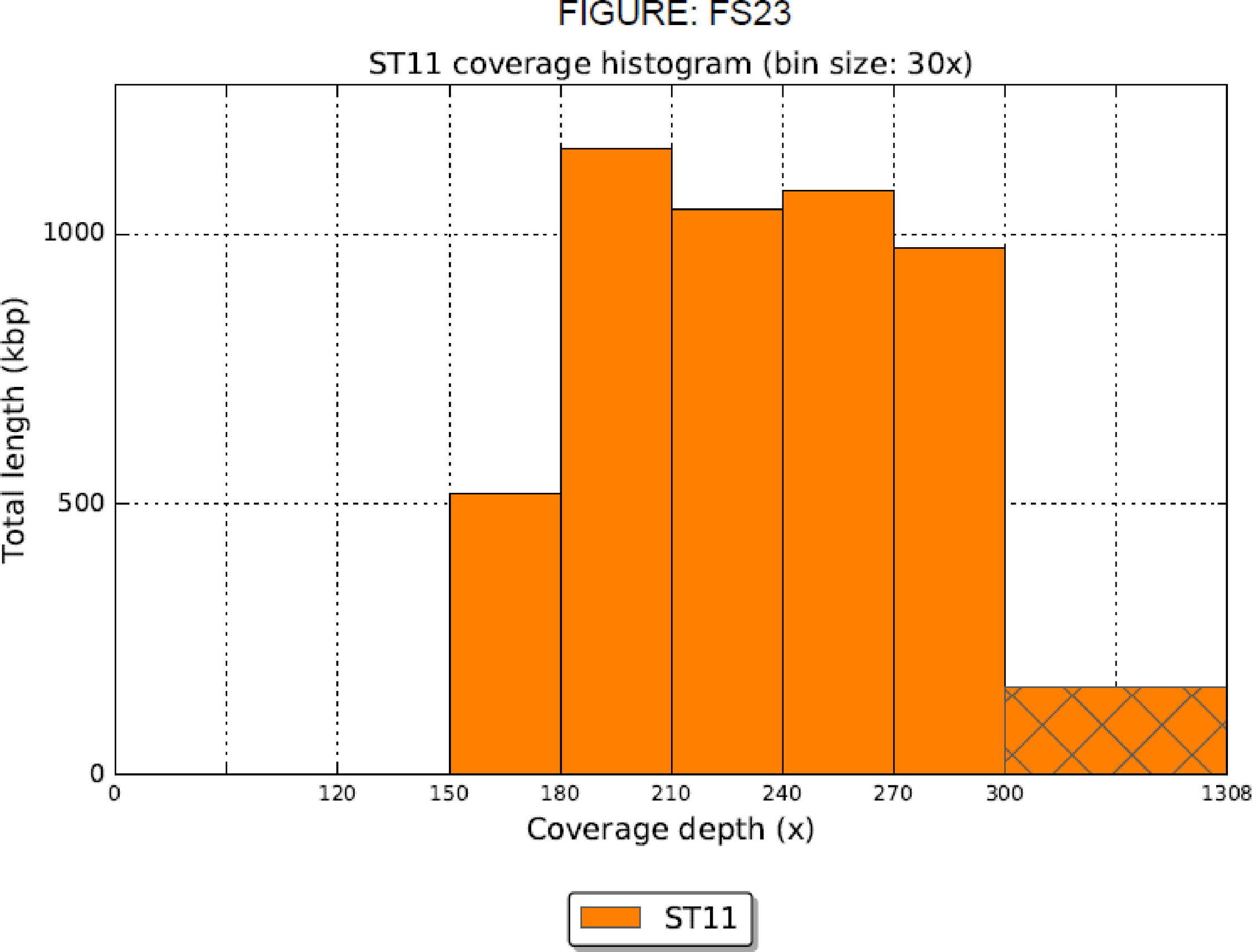

Supplement: S23 Fig — (TIF) [file pntd.0006839.s024.tif]

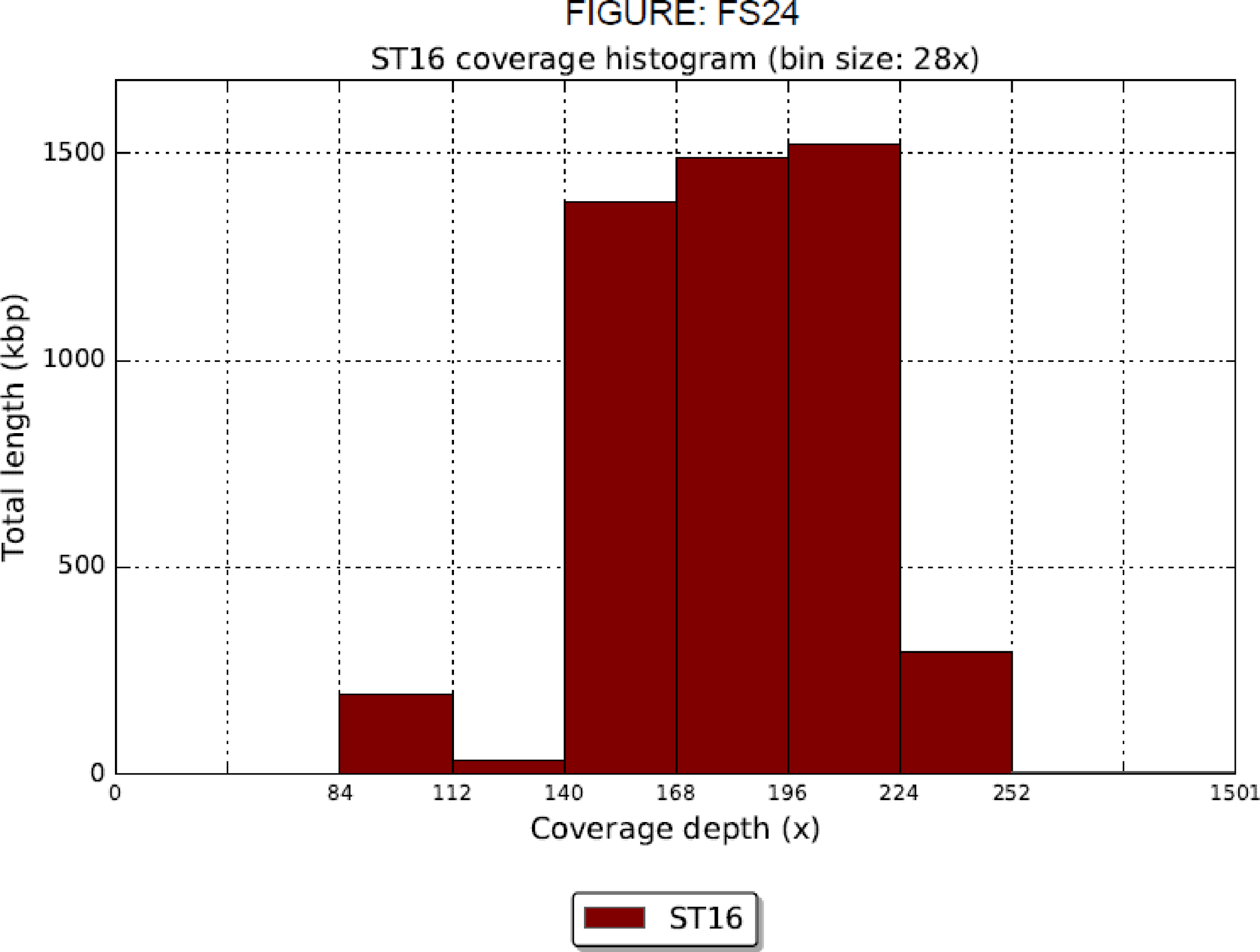

Supplement: S24 Fig — (TIF) [file pntd.0006839.s025.tif]

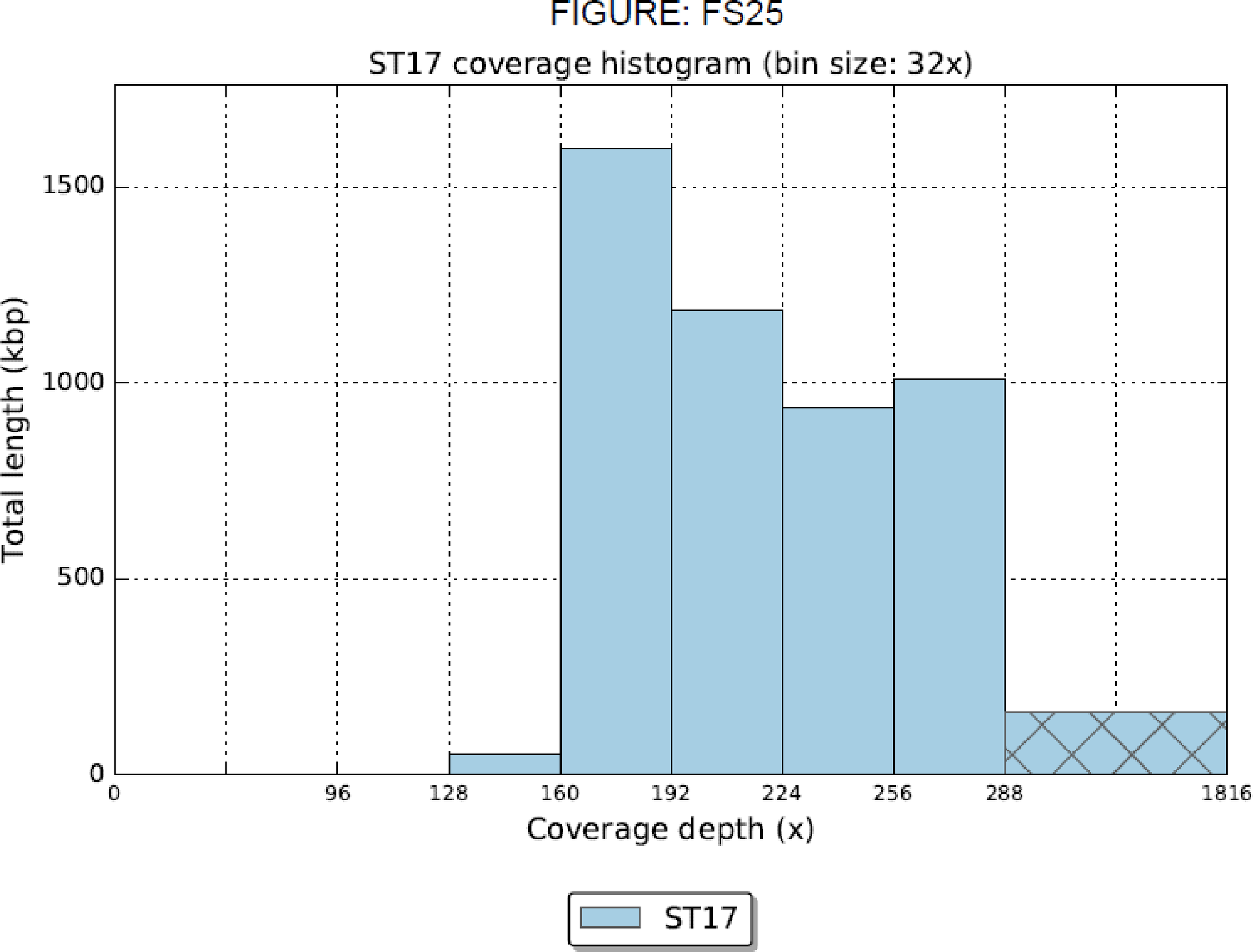

Supplement: S25 Fig — (TIF) [file pntd.0006839.s026.tif]

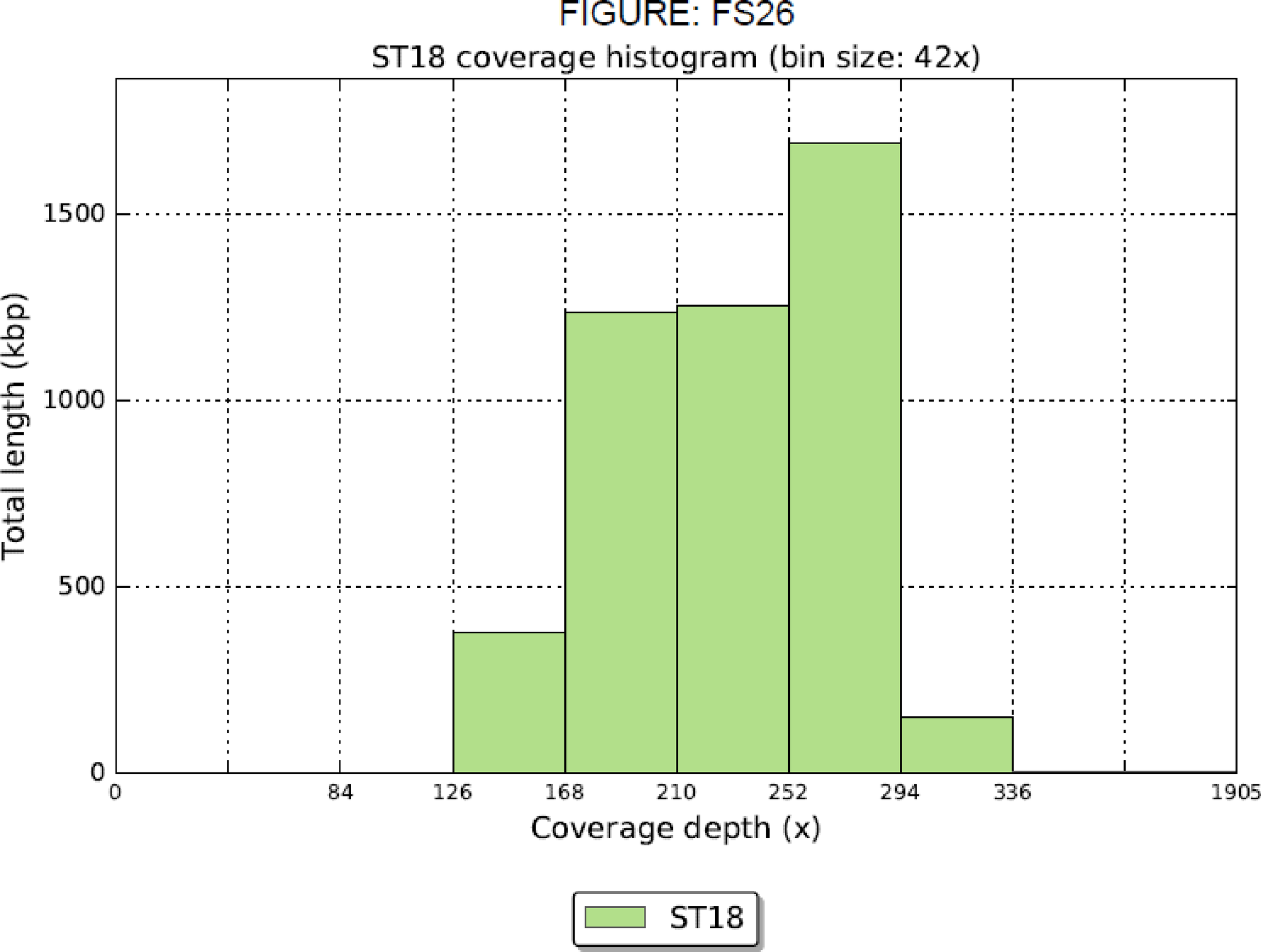

Supplement: S26 Fig — (TIF) [file pntd.0006839.s027.tif]

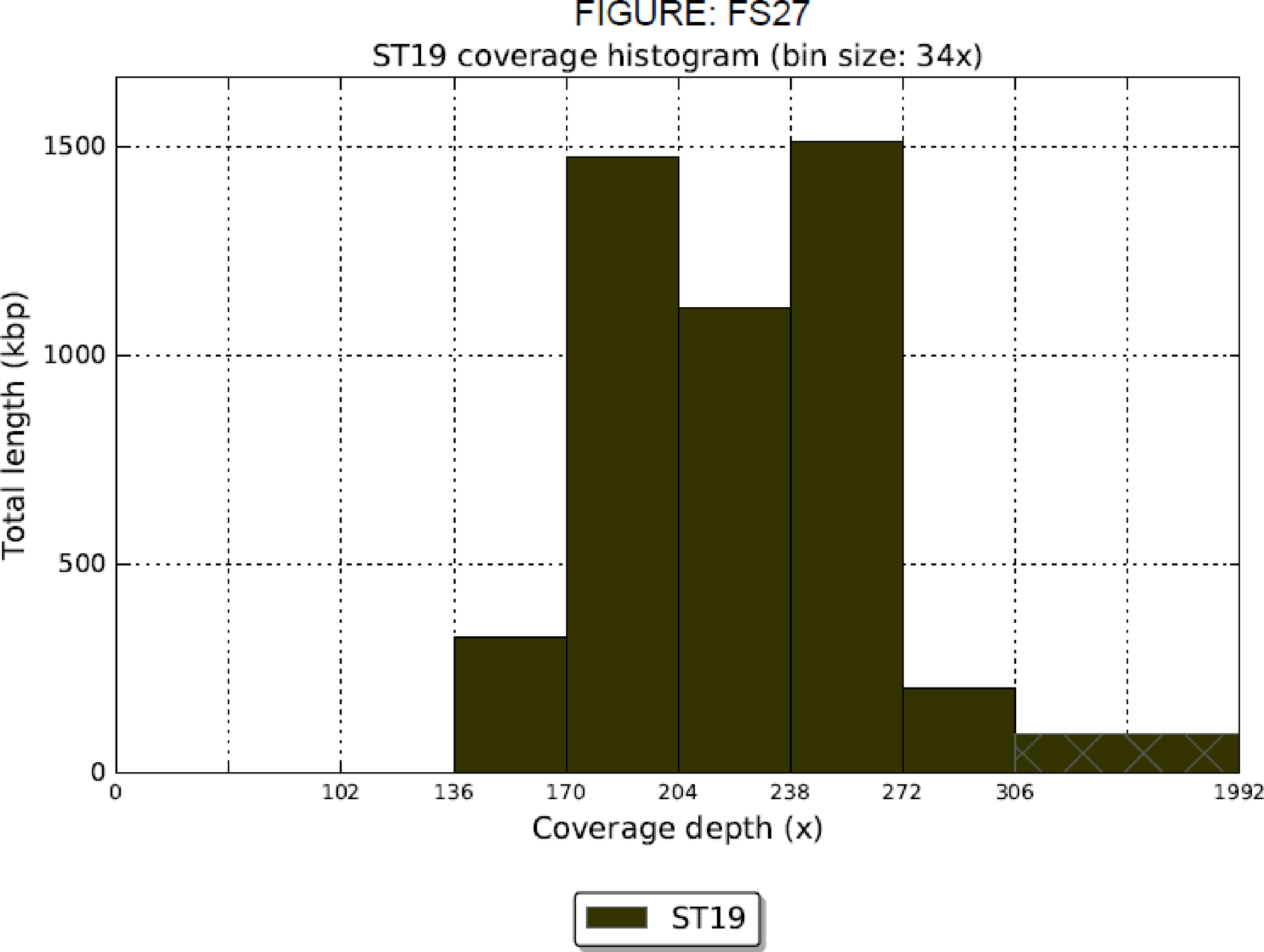

Supplement: S27 Fig — (TIF) [file pntd.0006839.s028.tif]

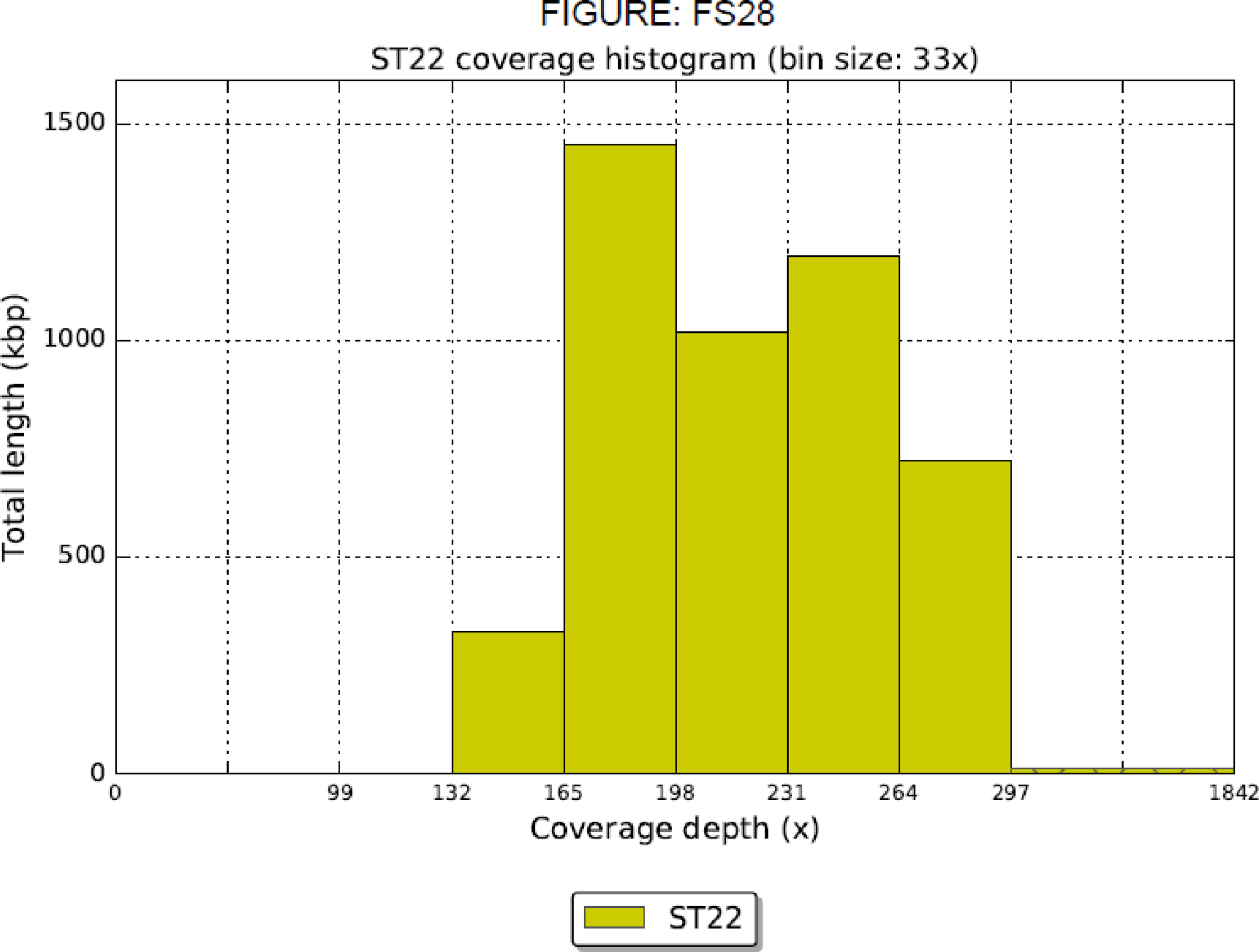

Supplement: S28 Fig — (TIF) [file pntd.0006839.s029.tif]

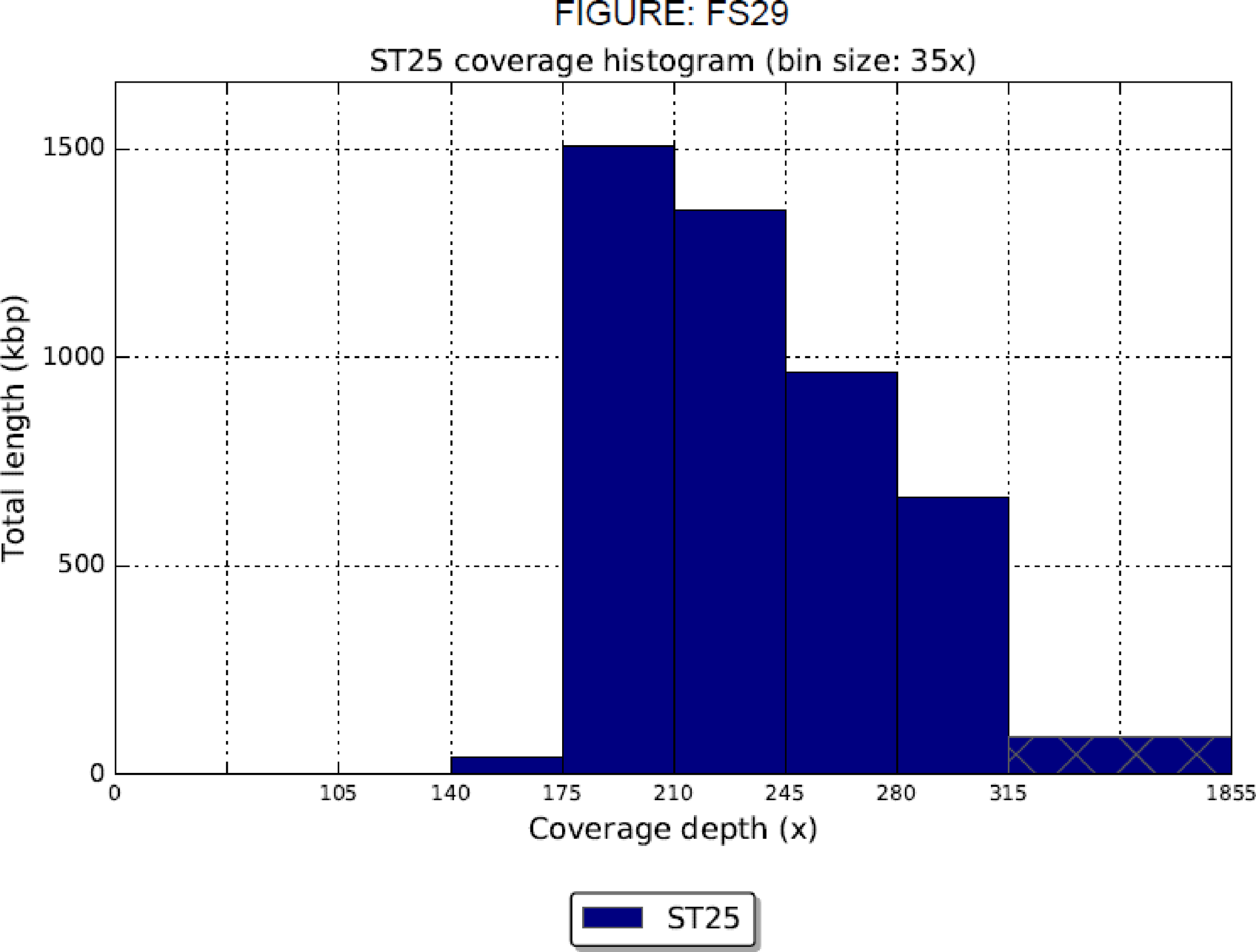

Supplement: S29 Fig — (TIF) [file pntd.0006839.s030.tif]

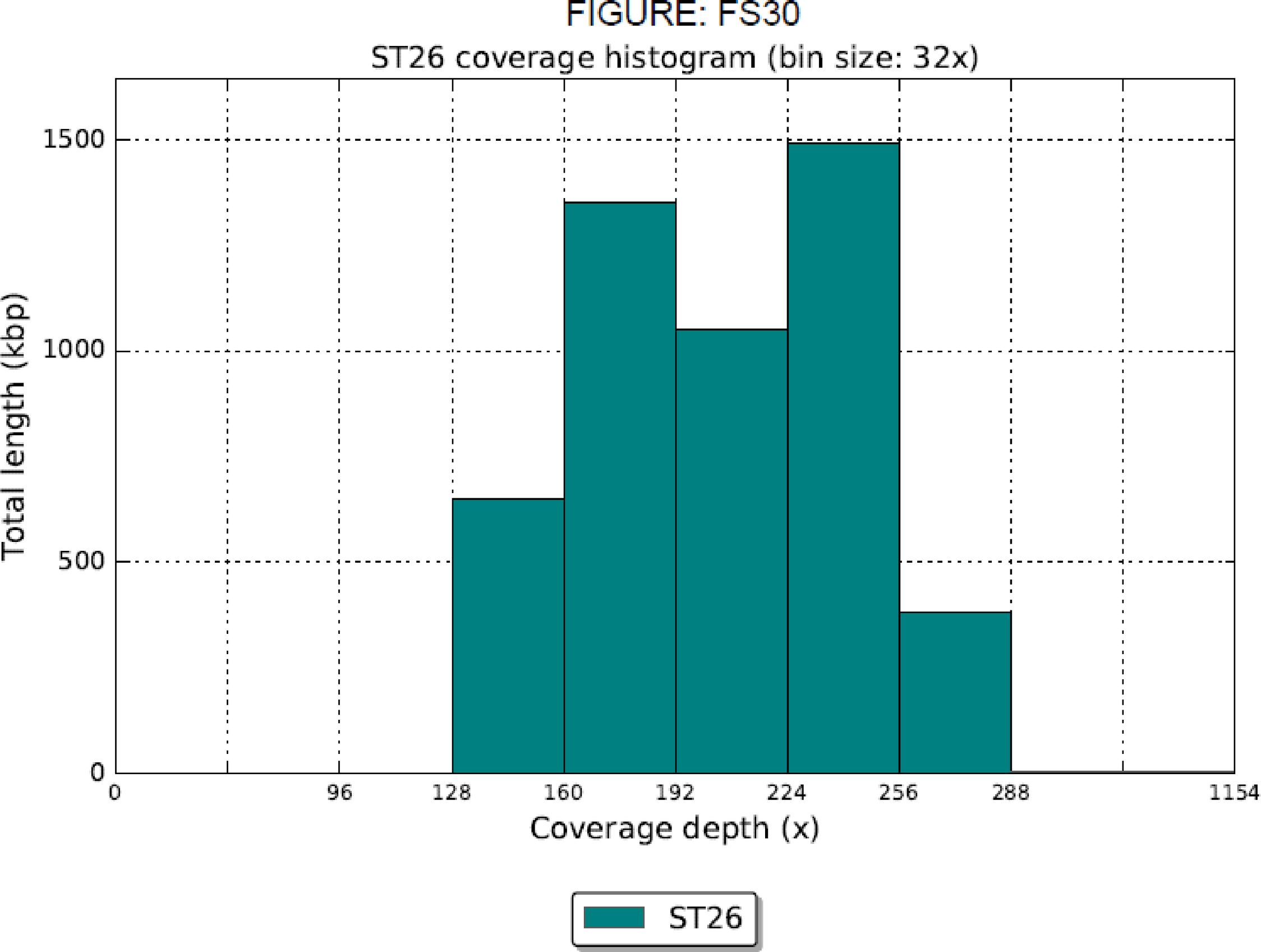

Supplement: S30 Fig — (TIF) [file pntd.0006839.s031.tif]

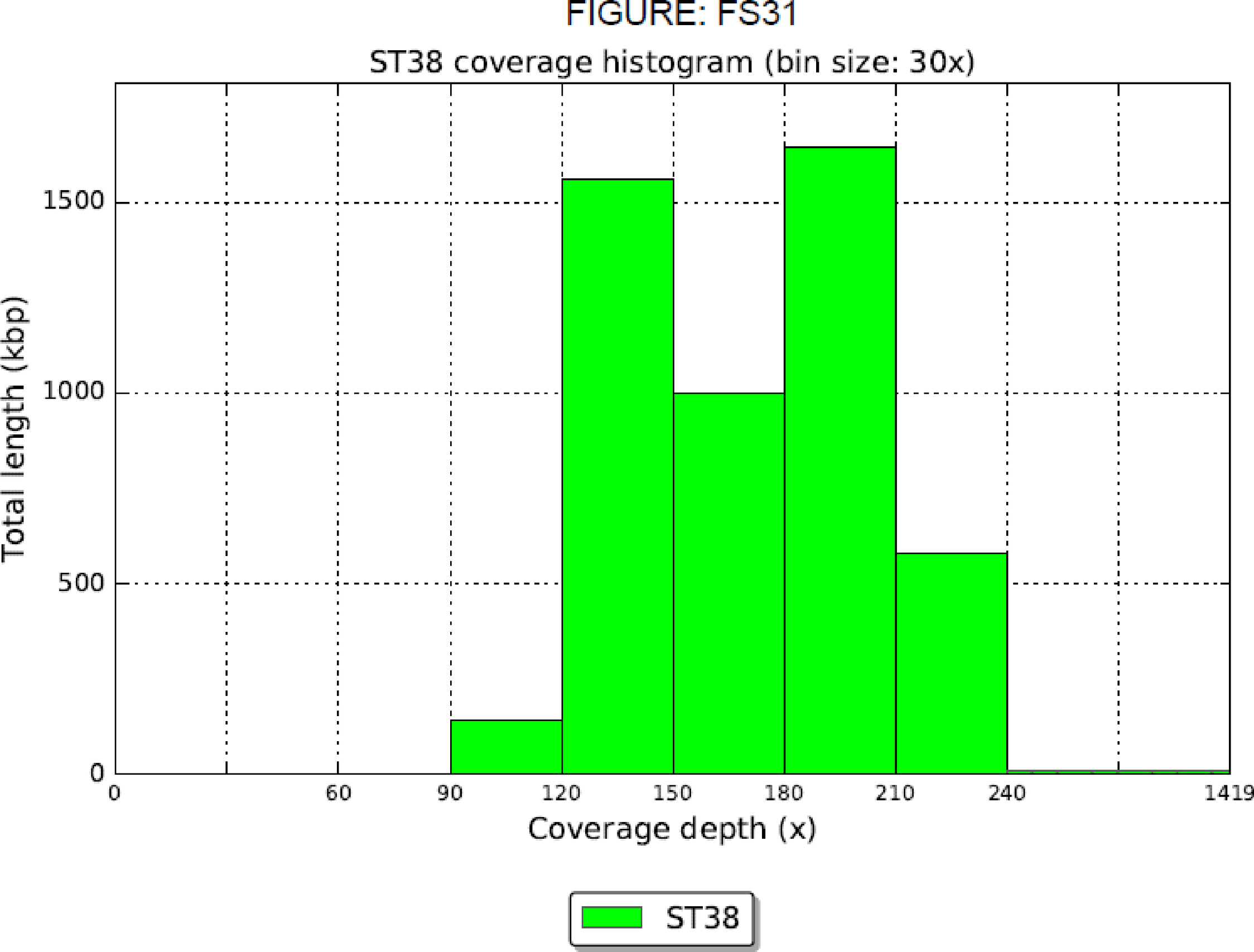

Supplement: S31 Fig — (TIF) [file pntd.0006839.s032.tif]
